# Multimodal assessment of exceptional responders refines subtyping-directed precision treatment in HR^+^/HER2^–^ breast cancer

**DOI:** 10.1172/JCI202305

**Published:** 2026-10-15

**Authors:** Yi Xiao, Fan Yang, Ying-Jia Qi, Li Chen, Hang Zhang, Si-Wei Zhang, Rui Shan, Liang Huang, Jun-Jie Li, Yi-Zhou Jiang, Zhi-Ming Shao

**Affiliations:** 1 Key Laboratory of Breast Cancer in Shanghai, Department of Breast Surgery, Fudan University Shanghai Cancer Center, Shanghai, China.; 2 Department of Oncology, Shanghai Medical College, Fudan University, Shanghai, China.; 3 Shanghai Academy of Natural Sciences (SANS), Fudan University, Shanghai, China.

**Keywords:** Cell biology, Clinical Research, Oncology, Bioinformatics, Breast cancer, Clinical trials

## Abstract

**BACKGROUND:**

We previously defined 4 hormone receptor^+^/human epidermal growth factor receptor 2^–^ (HR^+^/HER2^–^) subtypes: SNF1 canonical luminal, SNF2 immunogenic, SNF3 proliferative, and SNF4 RTK driven. The FASCINATE-N proof-of-concept trial demonstrated that subtyping-directed precision treatment improves pathologic complete response in locally advanced HR^+^/HER2^–^ breast cancer, yet efficacy remains suboptimal.

**METHODS:**

We integrated transcriptomic, genomic, digital pathology, and clinical data from this trial (*n* = 230) and focused on exceptional responders to explore opportunities for refining treatment strategies.

**RESULTS:**

In SNF1, we uncovered that perineural invasion diminished endocrine therapy efficacy, suggesting the potential benefit of incorporating perineural blockade. Digital pathology and spatial transcriptomics identified that the spatial architecture of the tumor-stromal-immune interface influenced apatinib efficacy in SNF4, highlighting the importance of interfering with the tumor microenvironment. In the SNF3 group, exceptionally well responders exhibited multiple mutations in DNA repair genes and cell cycle activation, correlating with the efficacy of poly (ADP-ribose) polymerase and CDK4/6 inhibitors. Additionally, we illustrated that lymphatic vessel infiltration was linked to improved immunotherapy outcomes in HR^+^/HER2^–^ SNF2 tumors.

**CONCLUSION:**

Overall, our study emphasizes the importance of multimodal assessments in optimizing precision treatments for HR^+^/HER2^–^ breast cancer.

**FUNDING:**

National Natural Science Foundation of China, grant numbers U25C2032, 82441028, and 82573645; The Shanghai Key Laboratory of Breast Cancer, grant number 12DZ2260100; The Human Phenome Data Center of Fudan University.

**TRIAL REGISTRATION:**

ClinicalTrials.gov identifier: NCT05582499.

## Abstract 2

This study focuses on exceptional responders in the FASCINATE-N trial to refine treatment strategies and optimize precision medicine for HR+/HER2- breast cancer

## Introduction

Hormone receptor^+^/human epidermal growth factor receptor 2^–^ (HR^+^/HER2^–^) breast cancer is the most prevalent subtype of breast cancer, accounting for approximately 70% of all breast cancer cases (1). Despite standard treatment, 25% of patients with early-stage disease later experience systemic recurrence (2). Although HR^+^/HER2^–^ breast cancer is characterized by considerable molecular heterogeneity, current treatment strategies remain relatively uniform, relying primarily on endocrine therapy, CDK4/6 inhibitors, and chemotherapy (3, 4). Through multiomics analysis, our group previously identified 4 distinct molecular subtypes of HR^+^/HER2^–^ breast cancer: the canonical luminal subtype (SNF1) may benefit from classic endocrine therapy; the immunogenic subtype (SNF2), enriched in immune cells, may benefit from immunotherapy; the proliferative subtype (SNF3), characterized by high homologous recombination deficiency (HRD) scores and cell cycle activation, may benefit from DNA-damaging agents and cell cycle inhibitors; and the RTK-driven subtype (SNF4), enriched in RTK pathway signatures, may benefit from RTK-related inhibitors. These subtypes exhibit unique biological and clinical characteristics, highlighting the potential for subtype-specific novel therapeutic approaches (5).

Platform trials play an important role in demonstrating proof of concept for those novel therapeutics, as they employ new clinical trial frameworks and statistical methods to accelerate drug research and development (6, 7), exemplified by trials such as I-SPY2, SHIVA, and STAMPEDE (8–10). I-SPY2 is a platform trial that uses a response-adaptive randomization within molecular subtypes to assess novel agents as neoadjuvant therapy for patients with early breast cancer. In the context of triple-negative breast cancer (TNBC), our group previously classified TNBC into 4 subtypes (11) and conducted the FUTURE platform trial to illustrate the promising efficacy of the subtyping-based precision strategy for patients with refractory metastatic TNBC (12). Given the success of subtyping-precision strategy in advanced breast cancer, we further extended its application to the neoadjuvant setting. The FASCINATE-N trial is a precision-based platform trial designed to evaluate the efficacy of subtyping-based treatments in patients with early-stage breast cancer undergoing neoadjuvant therapy (13). Despite a markedly higher pCR rate in the FASCINATE-N HR^+^/HER2^–^ cohort compared with the control arms, the overall pCR rate in locally advanced HR^+^/HER2^–^ tumors remains relatively low, at approximately 10%.

Therefore, it is crucial to focus on exceptional responders to identify effective biomarkers for well responders, while uncovering potential therapeutic targets for poor responders. This “exceptional responder” approach has been successfully utilized across various malignancies to generate critical insights into drug mechanisms and advance precision medicine, often despite limited sample sizes (14–18). The design of these prior studies aligns closely with the objectives and practical constraints of our FASCINATE-N cohort. However, exceptional responder analyses remain underexplored in HR^+^/HER2^–^ breast cancer, highlighting the necessity of our approach to optimize current subtype-directed treatments.

In this study, we established a multimodal cohort of HR^+^/HER2^–^ breast cancers derived from the FASCINATE-N trial. On the one hand, we emphasized the critical importance of SNF subtyping-based precision strategies for HR^+^/HER2^–^ breast cancers. On the other hand, we employed multimodal profiling of exceptional responders to identify key molecular features that distinguished well responders and uncovered additional therapeutic vulnerabilities for poor responders. Through this study, we aim to demonstrate that multimodal detection can enhance subtyping-directed precision treatments for HR^+^/HER2^–^ breast cancer.

## Results

### The FASCINATE-N HR^+^/HER2^–^ cohort and data resource: patients and data.

Our study is based on the HR^+^/HER2^–^ subgroup in the cohort of the FASCINATE-N trial, which conducted a precise molecular subtyping of HR^+^/HER2^–^ populations and tested subtyping-based precision treatment strategies (19). Based on the FASCINATE-N HR^+^/HER2^–^ cohort, a total of 230 patients with multiomics data for at least 1 dimension were included in this exploratory biomarker analysis (42 in SNF1, 48 in SNF2, 80 in SNF3, and 60 in SNF4). Subtyping-based precision treatment was applied in 141 patients: 25 patients with SNF1 tumors receiving dalpiciclib plus letrozole with goserelin if premenopausal; 26 patients with SNF2 tumors receiving nab-paclitaxel, carboplatin (PCb) plus adebrelimab; 25 patients with SNF3 tumors receiving PCb plus fluzoparib (arm 1) and 35 patients with SNF3 tumors receiving dalpiciclib, letrozole, and fluzoparib with goserelin if premenopausal (arm 2); and 30 patients with SNF4 tumors receiving PCb plus apatinib. Thirty-five patients with TNBC immunomodulatory (TNBC-IM) tumors receiving PCb followed by dose-intensive epirubicin and cyclophosphamide plus camrelizumab or control were also included to compare with the tumor microenvironment (TME) of SNF2 HR^+^/HER2^–^ tumors (Figure 1A and Supplemental Figure 1; supplemental material available online with this article; https://doi.org/10.1172/JCI202305DS1). Within this cohort of 230 patients with HR^+^/HER2^–^ breast cancer, the SNF subtyping-directed precision treatment yielded a higher pCR rate compared with traditional PCb treatment (11.3% vs. 4.5%), indicating a marked therapeutic advantage. However, while these findings support the efficacy of this subtyping-based strategy, the overall pCR rate remains modest, highlighting the need for further research to identify effective biomarkers and further explore therapeutic targets.

In order to conduct a more comprehensive analysis to further optimize current subtyping-based precision treatment strategies, we collected tumor tissue specimens, serial peripheral blood samples, and breast cancer histopathology whole-slide images (WSIs) of this cohort to establish a multiomics dataset (Figure 1B). This dataset consisted of bulk RNA-seq (RNA-seq 114, baseline 106, after 2 cycles 8), targeted sequencing (germline 163, somatic 153), WGS (*n* = 31), and 202 breast cancer histopathology WSIs (Figure 1C and Supplemental Table 1). In addition to the comprehensive analysis of each treatment arm, we focused on exceptional responders in the cohort to explore therapeutic clues, as the pCR rate in patients with HR^+^/HER2^–^ tumors was relatively low. We defined exceptional responders as 3 categories: well responders (pCR), poor responders (Miller-Payne [MP] grade ≤ 2), and extreme poor responders (MP grade ≤ 2 and evaluated as SD or PD during neoadjuvant treatment) (Figure 1D and Supplemental Table 1). The clinicopathological characteristics across different therapeutic efficacy within each SNF subtype are listed in Supplemental Table 2. By systematically analyzing the molecular profiles of these exceptional responders, we aim to uncover distinct resistance mechanisms and pinpoint actionable vulnerabilities that could effectively guide future personalized therapeutic interventions.

For each treatment arm, we first identify potential biomarkers within the overall population. Next, we evaluate the value of these biomarkers among exceptional responders. Finally, we substantiate our findings through external validation or in vivo experiments (Figure 1E).

### Expression of molecular biomarkers across the FASCINATE-N multiomics cohort.

First, we aimed to examine the distribution of molecular biomarkers across HR^+^/HER2^–^ subtypes generated by digital pathology–based AI models (5). In line with this objective, we gathered molecular biomarkers that were correlated with specific features in HR^+^/HER2^–^ tumors, including representative genes as well as pathways reflecting specific molecular features in the 4 SNF subtypes (Supplemental Figure 2). Then, we generated overall scores for each set of biomarkers in all SNF subtypes, supporting the high consistency between RNA-based biomarkers and AI-based subtypes (Figure 2A) (8, 20, 21). Specifically, the SNF1 and SNF3 subtypes, especially the SNF1 subtype, were characterized by the relatively high activation of ER pathways; the SNF2 subtype highly expressed biomarkers related to immune activation; the SNF3 subtype exhibited upregulation of cell cycle and DNA repair deficiency; and the SNF4 subtype was characterized by active RTK and VEGF signaling (Figure 2, A and B). Overall, these findings indicated that AI-based subtyping of HR^+^/HER2^–^ breast cancer could identify subpopulations potentially sensitive to specific treatments among patients with HR^+^/HER2^–^ breast cancers.

### GABAergic neurons undermine the effect of endocrine therapy for SNF1 HR^+^/HER2^–^ breast cancers.

Then, we utilized the multimodal data from each treatment arm to explore the determinants of efficacy. In addition to illustrating the multiomics landscape of each treatment arm, we concentrated on the characteristics of exceptional responders to explore clues for refining subtyping-directed precision treatment strategies in HR^+^/HER2^–^ breast cancer. The SNF1 subtype, characterized as the canonical luminal subtype, is known for its activation of ER pathways, which typically responds well to endocrine therapy. Forty-two SNF1 patients were enrolled, and 25 patients went into the precision treatment arm (Figure 3A). Of the precision treatment arm (dalpiciclib + letrozole ± OFS), 10 patients achieved partial response, while 15 patients were classified as exceptional responders (3 poor responders and 12 extreme poor responders).

Initially, RNA-seq analysis was conducted in this cohort to identify differentially expressed pathways between patients with favorable (partial response patients) and unfavorable (poor and extreme poor patients) outcomes. Notably, neuron-related pathways, particularly the GABA receptor pathway, were significantly upregulated in patients with poor efficacy, suggesting a role for perineural invasion (PNI) in endocrine resistance (Figure 3B). Particularly, neuron activation pathways were significantly upregulated in the extreme poor responders compared with poor responders (Figure 3C). In patient STE183, whose best therapy response assessment was SD, and MP stage was MP2, we found classical PNI structures within the tumor (Figure 3, D and E).

Then, we externally validated the clinical relevance of PNI in SNF1 patients from other cohorts. In our previously published HR^+^/HER2^–^ cohort (5), PNI was more prevalent in the SNF1 subgroup and was associated with a significantly worse prognosis, as confirmed through multivariate analysis (Figure 3, F and G; Supplemental Figure 3A; and Supplemental Tables 3 and 4). In the FELINE clinical trial (22), which involved patients receiving endocrine therapy (letrozole) plus the CDK4/6 inhibitor ribociclib, the GABA synthesis pathway was also activated in nonresponders (Figure 3H). Moreover, the neuron activation pathway was also upregulated in nonresponders at day 180 but not in responders (Figure 3I) (22). These findings aligned with the negative impact of PNI on the efficacy of neoadjuvant endocrine therapy observed in our clinical trial and suggested that combining endocrine therapy with neuroactive agents might be beneficial for patients whose tumors exhibit PNI.

To validate the function of GABAergic neurons in vivo, we established a GABAergic neuron ablation mouse model using resiniferatoxin (RTX) in BALB/c nude mice. RTX is known to ablate GABAergic fibers by inducing nerve terminal death (23). Immunofluorescence for GABA confirmed effective nerve ablation (Supplemental Figure 3B). Mice bearing MCF7 xenografts received endocrine therapy (tamoxifen) to mimic the clinical setting. Tumor growth was suppressed more significantly in mice treated with combined RTX and tamoxifen plus CDK4/6 inhibitor than in mice with tamoxifen plus CDK4/6 inhibitor alone (Figure 3, J and K, and Supplemental Figure 3C). In conclusion, we proposed that the presence of PNI constitutes a pivotal mechanism underlying the resistance to neoadjuvant endocrine therapy in SNF1 subtype (Figure 3L).

### Spatial structures influence the efficacy of apatinib in SNF4 HR^+^/HER2^–^ breast cancers.

SNF4 breast cancer, characterized by activation of the RTK signaling pathway, is prone to endocrine therapy resistance. Compared with other subtypes, patients with SNF4 exhibit markedly lower metastasis-free survival and recurrence-free survival, with an overall poorer prognosis. To uncover the potential factors affecting the efficacy of tyrosine kinase inhibitor (TKI) treatment in SNF4 breast cancer, 30 patients with SNF4 tumors were enrolled in the precision treatment arm, receiving neoadjuvant therapy consisting of TKI combined with PCb. Of these patients, 23 patients achieved partial response, while 7 patients were classified as exceptional responders (4 well responders, 2 poor responders, and 1 extreme poor responder). To investigate potential biomarkers of treatment efficacy, we collected tissue samples from these patients, each providing data for at least 1 modality for analysis (Figure 4A). RNA analysis of this cohort revealed that fibroblast-related pathways were enriched in the non-pCR group (Figure 4B), while immune-related pathways were enriched in the pCR group, suggesting that the composition and structure of the TME might influence apatinib efficacy.

Then, we systematically analyzed the TME composition and structure between the pCR and non-pCR groups. Our previous work established the sc-MTOP algorithm to identify cell types and spatial positions from the WSI (24). Therefore, we analyzed paraffin-embedded H&E tumor slides of this cohort to assess whether the composition and spatial characteristics of the TME contributed to the treatment response (Figure 4C and Supplemental Table 5). We observed that the pCR group tended to have a higher proportion of immune cells, whereas the non-pCR group tended to have a higher proportion of tumor cells (Figure 4D). Furthermore, patients achieving pCR exhibited more immune cell interactions with tumor cells, while non-pCR patients showed a higher prevalence of stromal barriers separating immune cells from tumor cells. This stromal barrier, or “stromaBlocker” feature, appeared to impede immune cell–tumor cell interactions in non-pCR patients (Figure 4, E and F). This pattern was particularly pronounced in SNF4 patients with exceptional response (Figure 4F). Specifically, 1 patient who achieved pCR after 4 treatment cycles showed notably close immune-tumor cell connectivity. In contrast, a non-pCR patient with an MP grade of 2 exhibited immune cells sequestered by stromal cells, limiting their interaction with tumor cells (Figure 4, G and H). To explore how spatial arrangement influences immune cell function, we analyzed public spatial transcriptomics data of an ER^+^ breast cancer sample (25). The H&E image was divided into distinct regions, with each region exhibiting unique markers and cell composition patterns (Supplemental Figure 4, A and B). Immune cells localized to stromal regions had lower activity of multiple immune-related pathways (Figure 4I and Supplemental Figure 4C), whereas intratumoral CD4^+^ T cells exhibited stronger predicted chemokine-mediated communication with cells expressing ACKR1, a chemokine receptor linked to immune activation and better prognosis (26) (Supplemental Figure 4D). Further high-plex spatial transcriptomic analysis using the CosMx SMI 6,175-gene panel, profiling 79,872 cells from 5 patients (Figure 4J), identified 2 T cell states: tumor-infiltrated T cells and stroma-blocked T cells (Figure 4K and Supplemental Figure 4E). Tumor-infiltrated T cells exhibited higher cytotoxic (e.g., GZMB, PRF1) and exhaustion (e.g., PDCD1, LAG3) markers, indicating an activated state, while stroma-blocked T cells displayed lower overall effector activity, linking stromal exclusion to immune suppression (Figure 4K). Spatial in situ imaging confirmed high GZMB expression in tumor-infiltrated T cells versus reduced expression in stroma-blocked T cells (Figure 4, L and M). This suggests that immune cells in the stromal region might experience functional impairment, while intratumoral immune cells actively engage in chemokine signaling, contributing to more effective immune responses.

In summary, the spatial architecture of the TME is correlated with the efficacy of precision treatment in SNF4. An immune-suppressive TME and collagen-enriched structures may contribute to the poor response to PCb combined with apatinib therapy. These findings provide a rationale for the preclinical evaluation of combining TKIs with stroma-targeting agents in SNF4 breast cancer (Figure 4N).

### Determinants of exceptional responses to poly (ADP-ribose) polymerase inhibitors combined with CDK4/6 inhibitors in SNF3 HR^+^/HER2^–^ breast cancers.

The SNF3 subtype of HR^+^/HER2^–^ breast cancer is known for high HRD level and high cell cycle activation; therefore, therapeutic arms targeting these features were designed in precision therapy. Here, we describe the characteristics of exceptional responders in SNF3 subtype and delve into the underlying mechanism of a well responder with a high HRD score. A total of 60 patients were enrolled in the precision treatment arm, receiving either a combination of CDK4/6 inhibitor dalpiciclib, poly (ADP-ribose) polymerase (PARP) inhibitor fluzoparib, and endocrine therapy, or neoadjuvant chemotherapy (PCb) combined with fluzoparib. Among them, 8 were well responders, 37 were partial responders, 7 were poor responders, and 8 were extreme poor responders. Tumor samples were collected from these patients, each providing data for at least 1 modality (Figure 5A).

We observed that patients exhibiting a favorable response to CDK4/6 inhibitor and PARP inhibitor had higher baseline HRD scores (*P* = 0.040) (Figure 5B), increased sensitivity scores for CDK4/6 inhibitors (*P* = 0.045) (Figure 5C), and greater activation of the base excision repair (BER) and cell cycle pathways compared with those with a poor or extreme poor response (Figure 5, D and E). Baseline Ki-67 scores were higher in well responders, indicating higher sensitivity to cell cycle inhibitors (Figure 5F). Moreover, among patients who underwent core needle biopsy following the second cycle of treatment (6 weeks), a general decline in Ki-67 scores was observed; however, while well responders typically transitioned to a nontumor state at this time point, Ki-67 scores in poor and extreme poor groups re-elevated at the time of surgery (18 weeks), suggesting evolving resistance to cell cycle inhibitors (Figure 5G). These results suggested that the activation of cell cycle may be indicative of a favorable therapeutic response to CDK4/6 inhibitors, while activation of single-stranded DNA repair pathway and deficiency of double-stranded DNA repair may signal sensitivity to neoadjuvant PARP inhibitors in patients with SNF3 breast cancer.

Particularly, this phenomenon was predominantly observed in a well responder, labeled STE068, in SNF3, who exhibited an exceptionally high HRD score (Figure 5, B and H). This patient harbored somatic mutations in 4 DNA repair–related genes, 3 of which are related to homologous recombination repair (HRR): *BRCA1*, which featured a nonsense mutation (c.2603C>G, p.S868X), *ATR* with a missense mutation (c.2550G>A, p.M850I), and *NSD2*, also with a missense mutation (c.3295G>A, p.E1099K). Additionally, a mutation was present in *XRCC5*, a gene related to nonhomologous end joining (NHEJ), characterized by a splice site mutation (c.369-1G>C). These mutations were located within functional domains, and the damaging effects of these mutations were predicted using various online tools, including CADD, PolyPhen-2, PROVEAN, and SIFT (Figure 5I and Supplemental Table 6). It was suggested that the remarkable therapeutic response of this patient was due to the damaging double-stranded DNA repair deficiency, encompassing both HRR and NHEJ, which exhibited synthetic lethality in conjunction with the pharmacological disruption of single-stranded DNA repair by PARP inhibitors (Figure 5J).

In conclusion, we found that the activation of single-stranded DNA repair, the deficiency of double-stranded DNA repair, and the activation of the cell cycle at baseline may indicate sensitivity to neoadjuvant PARP inhibitor in combination with CDK4/6 inhibitor in patients with SNF3 breast cancer, and the deficiency of double-stranded DNA repair caused by multiple damaging gene mutations may contribute to the well therapeutic response (Figure 5J).

### Key immune response patterns and predictive markers in SNF2 HR^+^/HER2^–^ breast cancers.

Building on prior findings that SNF2 subtype tumors are enriched with immune cells (5), we hypothesized that patients with the SNF2 subtype might exhibit sensitivity to immunotherapy. Twenty-six patients with HR^+^/HER2^–^ SNF2 tumors underwent neoadjuvant chemotherapy in combination with the anti–programmed cell death ligand 1 (anti–PD-L1) agent adebrelimab. Among these patients, 4 achieved pCR, and 18 were classified as partial responders, 1 as a poor responder, and 3 as extreme poor responders. Nineteen TNBC-IM subtype tumors were also included in the analysis. These patients received neoadjuvant chemotherapy along with camrelizumab, resulting in 11 well responders, 6 partial responders,1 poor responder, and 1 extreme poor responder (Figure 6A). Based on these therapeutic outcomes in SNF2 patients, we aimed to identify SNF2-specific treatment biomarkers for immunotherapy.

First, we systematically illustrated the TME features of the HR^+^/HER2^–^ SNF2 and TNBC-IM tumors. Strikingly, although the HR^+^/HER2^–^ SNF2 subtype is the most immunogenic among the HR^+^/HER2^–^ subtypes, it remained comparatively more “immune cold” than the TNBC-IM subtype (Figure 6B and Supplemental Table 7). The low abundance of immune cells partly explained why traditional immunotherapy biomarkers, such as CD8, PD-1, and PD-L1, are not valuable in the SNF2 arm (Supplemental Figure 5A). This situation drove us to further explore clinically accessible biomarkers for predicting the efficacy of neoadjuvant immunotherapy of the SNF2 arm. To achieve this aim, we analyzed the difference in signaling pathways between responders and nonresponders within the immunotherapy treatment arm of HR^+^/HER2^–^ SNF2 tumors. Tumors with favorable (well and partial) efficacy showed upregulated immune pathways but downregulated stromal pathways compared with tumors with unfavorable (extreme poor) efficacy (Figure 6C). Positive correlations among various immune cell types were also stronger within patients with favorable efficacy, while patients with unfavorable efficacy displayed insignificant or even negative correlations between immune cell types (Figure 6D). To identify the key changes of this immune stimulation, we focused on the immune pathways that were upregulated in the favorable compared with unfavorable efficacy groups, especially upregulated in the well subgroup compared with the extreme poor group. Interestingly, we found that the pathway associated with lymphatic vessels was upregulated in the favorable efficacy groups and even higher in the well subgroup compared with extreme poor group (Figure 6E). This enrichment suggested strong correlation between lymphatic vessels and immunotherapy efficacy. The key marker of lymphatic vessels, *LYVE1*, was highly positively correlated with a variety of immune activators, which indicated the high immune infiltration and active function facilitated by lymphatic vessels (Figure 6F and Supplemental Figure 5B). One patient, STE120, who achieved pCR, exhibited a remarkably high level of *LYVE1* and correspondingly higher levels of immune effector molecules, including *CD8A* and *PRF1*, suggesting the lymphatic vessel as a potential immunotherapy predictor. This immune relationship could be verified in SNF2 patients from an in-house retrospective cohort from CBCGA (Figure 6G and Supplemental Figure 5C) (27) and the HR^+^HER2^–^Immune^+^ patients from I-SPY2 (8) (Supplemental Figure 5D) but not in patients with the TNBC-IM subtype (Supplemental Figure 5, E and F). As a visible pathological structure, lymphatic vessels could be evaluated by pathologists on H&E-stained slides. All these findings suggested lymphatic vessels as a potential immunotherapy predictor specifically in the SNF2 subtype.

In general, HR^+^/HER2^–^ SNF2 tumors had a distinct TME from TNBC-IM tumors. The lymphatic vessel generation and the general immune activation were correlated with immunotherapy efficacy in HR^+^/HER2^–^ SNF2 patients (Figure 6H).

## Discussion

In this study, we introduced the FASCINATE-N Data Resource, a comprehensive compendium containing genomic, transcriptomic, and digital pathologic data for 230 patients with breast cancer in 5 subtyping-based precision arms within the FASCINATE-N HR^+^/HER2^–^ cohort (Figure 1). Our thorough evaluation demonstrated that the deep learning–based digital pathology classification system was clinically feasible for the subtyping and enrollment of patients with HR^+^/HER2^–^ tumors. Precision strategies were tailored to the molecular heterogeneity of the driving molecular events across the 4 SNF subtypes, leading to favorable outcomes (Figure 2). By multiomics analysis, we also uncovered mechanistic insights that explained differential responses to precision treatments across the subtypes (Figures 3–6). Our study provided compelling evidence supporting the efficacy of SNF subtyping-directed precision treatment strategies in improving outcomes for patients with HR^+^/HER2^–^ breast cancer and offered mechanistic hypotheses that could further refine these precision approaches.

Although the classification of molecular subtypes has been extensively utilized in oncology research, its translation into meaningful improvements in clinical management has been relatively rare. The I-SPY2 translational research created alternative breast cancer–subtyping schemes incorporating immune, DNA repair, and HER2/luminal phenotypes that more accurately predict drug responses and increase the pCR rate (8). However, the considerable financial and time constraints of those genomic tests limit their widespread application in clinical practice. We previously revealed heterogeneity among HR^+^/HER2^–^ breast cancers utilizing a deep learning–based digital pathology classification system (5). In this study, we verified that the digital pathology classification system was feasible and accurate, which provided a more comprehensive and accurate understanding of the specific subtype of patients with HR^+^/HER2^–^ breast cancer in a rapid and economical way (Figure 2). Importantly, rather than merely predicting pCR based on standard clinicopathological factors, the primary objective of our SNF subtyping is to elucidate underlying biological heterogeneity. This approach allows us to identify subtype-specific therapeutic targets and directly guide precision treatments. To rigorously evaluate the clinical benefit of our subtyping-based strategy, we selected the PCb regimen as the control arm. This decision was based on the fact that it enables safer combination with investigational agents.

Although the FASCINATE-N trial improved the pCR rate in HR^+^/HER2^–^ breast cancer following neoadjuvant therapy, the efficacy of targeted therapies in some therapeutic arms did not meet expectations. In our study, we observed that the enrichment of GABA synthesis was indicative of endocrine resistance in SNF1 subtype (Figure 3). Increased GABA synthesis within tumors can contribute to an immunosuppressive microenvironment and foster tumor growth (28). In the nervous system, estradiol-ER interactions in hypothalamic neurons lead to presynaptic GABA release (29). We identified GABA synthesis as a substantial biomarker of endocrine resistance in the neoadjuvant setting and propose that a GABA-targeting approach, in combination with tamoxifen, may benefit patients with SNF1 subtype tumors. The SNF4 subtype is characterized by endocrine therapy insensitivity and poor prognosis and represents a considerable unmet medical need. Previous clinical trials have shown that apatinib, when combined with other treatments, is effective and tolerable in advanced breast cancer (30, 31). In our study, apatinib demonstrated the ability to inhibit cancer-promoting stromal cells and reduce tumor growth in SNF4 tumors. Additionally, at the spatial level, we uncovered a mechanism contributing to immune suppression within SNF4 tumors, where immune cells in the stromal regions exhibited reduced activity. ACKR1, an atypical chemokine receptor involved in chemokine binding and immune cell trafficking (32), showed disrupted cell communication in SNF4 tumors, impairing interactions between CD4^+^ T cells and tumor cells and potentially contributing to immune evasion and apatinib resistance. Unlike in TNBC, the efficacy of PARP inhibitors in BRCA1/2-mutated HR^+^/HER2^–^ breast cancer remains poorly understood, highlighting the need for further investigation into biomarkers to better predict PARP inhibitor response (33). Combining TKIs with stroma-targeting agents, such as TGF-β inhibitors, represents a promising strategy for SNF4 patients. Such agents could degrade the dense extracellular matrix and normalize the stroma, thereby promoting the intratumoral infiltration of T lymphocytes and sensitizing tumors to treatment. Consistent with expectation, we found that a higher HRD score and a higher CDK4/6 inhibitor sensitivity score were associated with improved prognosis. In addition, patients who exhibited an excellent therapeutic response demonstrated enrichment of the BER pathway and mutations in homologous recombination repair-related genes. PARP1 plays a crucial role in the BER pathway by catalyzing the addition of ADP-ribose units to form poly (ADP-ribose) chains, which signal and recruit key components of the BER machinery responsible for repairing small, non-helix-distorting lesions in DNA (34). This finding suggests that the BER pathway may play a more pivotal role than NHEJ or direct repair pathways in HR^+^/HER2^–^ breast cancer. HR^+^/HER2^–^ breast cancer is well known for its lack of tumor-infiltrating lymphocytes and PD-L1 expression and is therefore considered “immune-cold” (35). However, we found a mechanism of immunotherapy resistance in SNF2 tumors that was absent in TNBC, namely lymphatic vessels. Lymphatic vessels’ dual roles in promoting metastasis and modulating immune dynamics may help explain the distinct importance of lymphatic vessels in TNBC versus HR^+^/HER2^–^ breast cancer (36).

One of the limitations of this research is its small sample size and single-center setup. Continuous, long-term follow-up is necessary to fully assess clinical outcomes, as the use of pCR as an endpoint in the HR^+^/HER2^–^ neoadjuvant setting remains a topic of debate. Nevertheless, this result of the FASCINATE-N HR^+^/HER2^–^ cohort is encouraging. Compared with control groups, the subtyping-based precision strategy improved the outcome of HR^+^/HER2^–^ breast cancer, particularly for patients with SNF3 and SNF4 tumors. Plans are underway to conduct additional studies with larger cohorts and well-designed head-to-head comparisons. Moreover, we will further explore and validate our findings through ongoing monitoring of long-term survival indicators, such as event-free survival. Another limitation of our research is that our mechanistic findings derive from a small number of well or poor responders. This constraint would inevitably introduce potential disadvantages, including susceptibility to selection bias, reduced generalizability to broader populations, and the risk of overinterpreting specific findings. However, we addressed this limitation by conducting external validation and experimental confirmation of our findings. Finally, as our current study primarily focused on baseline molecular profiling to guide neoadjuvant therapy, the potential impact of treatment-induced subtype plasticity and phenotypic switching remains to be fully characterized. Comprehensive longitudinal monitoring is required in future studies to elucidate mechanisms of resistance and refine adaptive treatment strategies.

## Methods

### Sex as a biological variable

All human participants in our cohorts were female, which is representative of the patient population for breast cancer, a disease that predominantly affects females. For the in vivo studies, only female mice were used to establish orthotopic tumor models.

### FASCINATE-N trial HR+/HER2– cohort overview

FASCINATE-N is a randomized, open-label, umbrella trial to evaluate the efficacy of targeted therapies in the neoadjuvant treatment of breast cancer according to subtypes (NCT05582499) (13). It is a platform comparing the efficacy of novel drugs in combination with standard chemotherapy with the efficacy of standard therapy alone and identifying improved treatment regimens for subsets based on clinical subtyping. A key feature of the trial is the Bayesian adaptive design, allowing independent analysis and reporting of individual arms upon meeting predefined success or termination criteria. Additionally, the trial protocol was structured to support both primary clinical evaluations and integral translational research (19). Within this broad umbrella framework, specific treatment arms were established to evaluate targeted therapies for patients with HR^+^/HER2^–^ breast cancer. This study specifically focuses on these HR^+^/HER2^–^ cohorts, and it presents the corresponding, prospectively planned translational study within the 230 patients with multiomics data for at least 1 dimension. The primary clinical endpoints for the HR^+^/HER2^–^ arms have been reported in detail elsewhere (19).

The primary endpoint was a pCR, defined as pathological stage (after neoadjuvant, abbreviated yp) ypT0/Tis ypN0, determined by a local pathologist at the time of surgery. Patients who switch to nonprotocol therapy, progress, forgo surgery, or withdraw are deemed “non-pCR.” HR^+^/HER2^–^ was defined as HR-positive (IHC ER > 1% and/or IHC PR > 1%) and HER2-negative (IHC 0/1+, or IHC 2+ with negative fluorescence/chromogenic in situ hybridization).

### Study design and treatment of FASCINATE-N HR+/HER2– cohort

The FASCINATE-N trial was prospectively designed to support both primary clinical investigations and integral translational research. This current study represents the translational component of the trial, focusing specifically on the cohort of patients with HR^+^/HER2^–^ breast cancer to uncover molecular features associated with therapeutic response and resistance.

The detailed workflow for enrolled patients was as follows. Initially, HR^+^/HER2^–^ tumors were classified into 4 subtypes: SNF1, SNF2, SNF3, or SNF4. Digital pathology subtyping was performed on biopsy samples from breast tumors at the Department of Pathology of FUSCC and Key Laboratory of Breast Cancer.

Based on biological characteristics of different subtypes, treatment groups are preliminarily set, then subdivided into precision treatment cohorts, and control cohorts are simultaneously set. Ten to thirty patients are enrolled in each precision treatment cohort. There were already 5 precision treatment cohorts in HR^+^/HER2^–^ cohort, and new precision treatment cohorts may also be added subsequently.

Participants were categorized into subtypes and randomly assigned to receive either subtype-specific precision therapy or control (nab-paclitaxel [100 mg/m^2^ i.v. day 1, day 8, and day 21 in a 28-day cycle] combined with carboplatin [AUC 1.5 i.v. day 1, day 8, and day 21 in a 28-day cycle]). We selected the PCb regimen as the chemotherapy backbone to ensure a favorable safety profile when combined with investigational agents. While EC-T (epirubicin and cyclophosphamide followed by taxanes) is considered the gold standard, the cumulative toxicity associated with epirubicin precludes its safe combination with the investigational agents used in this study. Patients with SNF1 tumors were randomized 1:1 to receive dalpiciclib (150 mg orally once daily for 3 consecutive weeks for 1 cycle), letrozole (2.5 mg orally once daily), and goserelin (3.6 mg i.m. every 4 weeks, for premenopausal patients) for 6 cycles or standard chemotherapy. Patients with SNF2 tumors were randomized 1:1 to receive standard chemotherapy with or without adebrelimab (10 mg/kg i.v. once every 2 weeks) for 6 cycles. Patients with SNF3 tumors were randomized to receive either standard chemotherapy for 6 cycles or precision therapy (arm 1: fluzoparib 50 mg p.o. twice daily per 28-day cycle, letrozole 2.5 mg p.o. once daily, dalpiciclib 150 mg p.o. once daily, and goserelin 3.6 mg i.m. every 4 weeks for premenopausal patients; arm 2: standard chemotherapy combined with fluzoparib 50 mg p.o. twice daily per 28-day cycle) for 6 cycles. Patients with the SNF4 tumors were randomized 1:1 to receive standard chemotherapy with or without apatinib (250 mg p.o. once daily) for 6 cycles. After completion of neoadjuvant therapy, patients underwent breast surgery with axillary node surgery.

### TNBC IM cohort overview

To explore the differences in response to immunotherapy between HR^+^/HER2^–^ breast cancers and TNBC, we also included the TNBC IM cohort in the FASCINATE-N trial. TNBC tumors are stratified using FUSCC classification based on immunohistochemical markers androgen receptor (AR), CD8, and FOXC1 as IM (AR^–^ and CD8^+^) (37). Patients with IM tumors were randomized 1:1 to receive nab-paclitaxel (100 mg/m^2^ i.v. day 1, day 8, and day 21 in a 28-day cycle) and carboplatin (AUC 1.5 i.v. day 1, day 8, and day 21 in a 28-day cycle) for 4 cycles followed by sequential epirubicin (80–90 mg/m^2^ i.v. q2w) and cyclophosphamide (500 mg/m^2^ i.v. q2w, dose-intensive administration, once every 2 weeks) for 4 cycles with or without camrelizumab (200 mg/dose i.v. once every 2 weeks for 12 doses). After completion of neoadjuvant therapy, patients underwent breast surgery with axillary node surgery. In this study, we included 35 patients from the TNBC IM cohort with at least 1 dimension of multiomics data, for comparison with the HR^+^/HER2^–^ SNF2 tumors treated with chemotherapy with or without immunotherapy.

### Specimen collection and processing

We performed comprehensive omics sequencing on all available clinical samples without bias. Core needle biopsy samples and peripheral blood samples were collected at baseline and after 2 treatment cycles. The surgically resected tissues were collected within 30 minutes after surgery. Collected tissue samples by core biopsies and surgery were immediately frozen and then stored at –80°C until further processing. Peripheral blood samples were stored at –80°C. Freshly frozen tumor tissues were subjected to macrodissection to minimize the potential influence of stromal tissues, thereby ensuring the presence of 50% or more tumor cells in all breast cancer specimens. The FFPE tissue sections were protected from light and moisture at the tissue bank. Additionally, some tumor samples were stored in RNAlater (Invitrogen) solution at –20°C. All the samples collected were stored at the FUSCC. Each sample has corresponding detailed clinical information.

We purified the genomic DNA from fresh-frozen samples and peripheral blood cells using TGuide M24 (Tiangen). The assessment of DNA purity and quantity involved measuring the absorbance at 260 nm (A260) and 280 nm (A280) using a NanoDrop 2000 spectrophotometer (Thermo Fisher Scientific). The extracted DNA with an A260/A280 ratio ranging from 1.6 to 1.9 was regarded as pure and suitable for subsequent experimental procedures. According to the manufacturer’s instructions, we purified the total RNA from tissues that had been previously stored in RNAlater solution using the miRNeasy Mini Kit (QIAGEN, 217004). The integrity of RNA was assessed using an Agilent 4200 Bioanalyzer alongside RNA ScreenTape (Agilent), while concentrations were determined by a NanoDrop ND-8000 spectrophotometer (Thermo Fisher Scientific).

### Generation and analysis of multiomics data

#### RNA-seq.

The ribosomal RNA was depleted using a Ribo-off rRNA Depletion Kit (Vazyme N406) from total RNA, and libraries were constructed using a VAHTS Universal V8 RNA-seq Library Prep Kit for Illumina (Vazyme NR605) involving the steps of fragmentation, RNA reverse transcription, poly(A) modification, and adapter attachment. Having evaluated the quality and quantity, paired-end sequencing was conducted on the Illumina NovaSeq platforms. Raw Illumina data underwent demultiplexing and conversion to FASTQ files, with subsequent quantification of adapter and low-quality sequences. HISAT2 was used to map reads to the GRCh38 reference genome. FPKM values of expressions were obtained through StringTie and Ballgown, and we discarded genes with an FPKM of 0 in more than 30% of the samples.

### WGS

#### DNA extraction.

For fresh-frozen tissue total DNA was extracted from the 10 mg tissues using QIAamp DNA Mini Kit (QIAGEN-51306) according to the manufacturer’s instructions. For paired peripheral blood mononuclear cells, total DNA was extracted from 1 mL blood using QIAamp DNA Blood Mini Kit (QIAGEN-51106) according to the manufacturer’s instructions. Then the integrity and concentration of the total DNA were determined by agarose electrophoresis and Qubit 2.0 fluorometer dsDNA HS Assay (Thermo Fisher Scientific). OD260/OD280 was measured by NanoDrop 2000 (Thermo Fisher Scientific).

#### Library preparation.

About 50 ng of high-quality genomic DNA was sheared with Covaris LE220 Sonicator to about 350 bp. The library was constructed according to the protocol of the KAPA Hyper Prep kit (Roche). First the fragmented DNA was purified using sample purification beads, the product was repaired by the end, and A base was added to the 3′ end. Then, the adapters were ligated with the specific barcode sequence. The CleanNGS magnetic beads (CleanNA) were used to screen out incomplete connections and self-connecting products. Sequencing libraries were formed by PCR amplification using universal primers complementary to the adaptor sequences.

#### Library quality control.

After library construction, the Qubit 3.0 fluorometer dsDNA HS Assay (Thermo Fisher Scientific) was used to quantify the concentration of the libraries, while the size distribution was analyzed by Agilent Bioanalyzer 4200.

#### Sequencing.

Paired-end sequencing was performed using the MGI DNBSEQ-T7 RS FCL DNA library prep set V3.0 (PE150) on the MGI DNBSEQ-T7 platform (Shenzhen, China) by Sequanta technologies (Shanghai, China) according to the manufacturer’s instructions.

#### Somatic mutation calling.

Raw sequence data were first aligned to the human reference genome GRCh38 (https://gdc.cancer.gov/about-data/gdc-data-processing/gdc-reference-files) using Burrows-Wheeler Aligner (BWA; version 0.7.17) with default parameters. For each paired sample, somatic single nucleotide variants and indels were detected by Sentieon TNscope (version 202010.01). Furthermore, in order to filter out germline variants, we generated a generic panel of normal (PON) file using the matched normal samples. Mutations in low-complexity regions, such as tandem repeat regions and highly homologous regions in the genome, were filtered out. Low-confidence variants were removed if any 1 of the following criteria was not satisfied: alternative allele depth in tumor ≥ 3, variant allele frequency in tumor ≥ 0.05 (if a mutation is annotated as a Catalogue Of Somatic Mutations In Cancer database somatic mutation, alternative allele depth in tumor ≥ 2, variant allele frequency in tumor ≥ 0.03), and variant allele frequency in normal/variant allele frequency in tumor < 0.2. Finally, all high-confidence mutations were then annotated with ANNOVAR (version 20200607) (38).

### Sequencing using the FUSCC-BC panel

A custom-designed genetic panel, comprising a hybridization-capture-based assay of 484 genes that are targets of approved and experimental therapies and frequently mutated genes in breast cancer, was used in this study. The panel was designed for the detection of mutations and small indels. In-house-generated RNA baits were utilized to capture all protein-coding exons of the target genes. The RNA baits were generated from an oligo pool synthesized by Synbio Technologies. The oligo pool was amplified into double-stranded DNA. Then, a T7 promoter site was incorporated into the amplicon, and the DNA was transcribed into biotinylated RNA. The biotinylated RNA was purified, quantified, and prepared for target enrichment.

Both the tumor and matched blood samples were sequenced. At least 10 ng of each DNA sample obtained after SYBR green quantification was fragmented using a Covaris M220 and then subjected to end repair, A-tailing, and adapter ligation using a KAPA HyperPlus kit (KAPA Biosystems) according to the manufacturer’s recommended protocol. Subsequently, 750 ng of prepared DNA in a volume of 3.4 μL was captured by RNA baits, and then, the captured library was purified and amplified using index primers. After quantification with a Multi-Mode Reader (BioTek), the libraries were pooled and sequenced using an Illumina HiSeq X TEN platform.

Data were collected using Illumina Real Time Analysis and assembled to FASTQ files using Illumina Bcl2Fastq2. The variant calling and coverage analysis of each capture region were performed using an in-house-developed bioinformatics pipeline based on the general variant calling pipeline. Briefly, the high-quality reads were mapped to the hg19 version of the human reference genome (GRCh37) using the BWA with the BWA-MEM algorithm and default parameters. The Genome Analysis Toolkit (GATK) was used to locally realign the BAM files at intervals with mismatched indels and recalibrate the base quality scores of the reads in the BAM files. Germline variants from the blood BAM file were identified using GATK HaplotypeCaller. Somatic mutations were called from the tissue and blood BAM files using GATK4 Mutect2 with the default parameters. The VCF files were annotated using ANNOVAR. The variants and annotation results were transferred into Microsoft Excel spreadsheets. To improve the specificity, a PON sample filter was used to filter the expected germline variations and artifacts (11). Each alteration identified by the pipeline was manually reviewed to ensure that no false positives were reported. The sequencing quality statistics were obtained using SAMtools and GATK. FACETS algorithm was utilized in detecting gene-level amplification and deletion.

A modified version of the Characterization of Germline Variants (CharGer), an automated scoring system according to the guidelines provided by the American College of Medical Genetics and Genomics, was used to further classify germline variants (39, 40). First, as the current version of CharGer does not include minor allele frequency (MAF) data for the East Asian population, we manually filtered our candidate variants according to the Genome Aggregation Database. Only rare variants (MAF < 0.5% in the East Asian population) were retained. Second, we manually updated the ClinVar records, and only variants known to be related to cancer were kept for PS1 and PM5 classifications. As we disabled the PM2 module, we slightly adjusted the cutoff values used for variant classification: variants with a CharGer score higher than 7 were considered likely pathogenic mutations, and variants known to be pathogenic were classified as pathogenic mutations.

### Digital pathology

Nuclear morphological features and texture features were extracted for each cell. Morphological features describe nuclear area, perimeter, elongation, and curvature. Texture features characterize features on the basis of gray-level co-occurrence matrix and statistics of pixel intensity within the nuclear contour.

Topological features were extracted to characterize the intercellular relationship at the single-cell resolution through a graph-based method proposed by us (24). For each pair of cell types, a graph was constructed denoted as Graph_C1-C2, where C1 and C2 denoted the cell types. We focused on tumor, inflammatory, and stroma cells. For the construction of each graph, k-nearest neighbor algorithm was used for edge configuration. Each cell is connected to its 5 nearest cells according to the Euclidean distance, and the edges longer than a threshold of 100 pixel (25 μm) are deleted. For each cell, the topological features were computed separately for each graph as it appeared and were concatenated to constitute its final comprehensive topological features.

The nuclear features and topological features for patients were obtained by averaging the relevant features of each cell type. The features are described in Supplemental Table 5.

### Generation and analysis of predictive biomarkers

#### Estimation of HRD scores.

The HRD score was the sum of the following 3 independent scores: telomeric allelic imbalance (TAI) score, loss of heterozygosity (LOH) score, and large-scale state transitions (LST) score. All these scores were calculated using mutation data derived from WGS based on a weighted model called HRDetect (41). Briefly, the TAI score represented the count of subchromosomal regions longer than 11 Mb and exhibited allelic imbalance extending to the telomere. The LOH score was calculated as the number of LOH regions longer than 15 Mb but shorter than the whole chromosome (LOH regions located on chromosome 17 were excluded). The LST score was defined as the number of breakpoints between 2 chromosomal regions longer than 10 Mb after regions shorter than 3 Mb were smoothed out.

#### Generation of multiple molecular biomarkers.

The gene signatures Module3_IFN, Module5_TcellBcell, Module7_ERBB2, Module10_ECM, and Module11_Prolif were referenced from Wolf et al. (20). The B_cells, Dendritic_cells, and Mast_cells signatures were derived from Danaher et al. (21). ESR1_PGR_ave and VCpred_TN were based on Wolf et al. (8).

Except for VCpred_TN, biomarkers based on the expression of multiple genes were calculated using the following steps: (a) mean centering of gene expression values, (b) averaging over the relevant genes, and (c) conversion into *Z*-scores for standardization (8).

Additionally, we downloaded gene sets from the MSigDB database (https://www.gsea-msigdb.org/gsea/msigdb) as complementary biomarkers to characterize the HR^+^/HER2^–^ breast cancer.

The generation of subtype-specific feature scores, including estrogen response, immune, HRD, and RTK and VEGF scores, involved 2 steps. First, pathways or molecules indicative of specific SNF subtypes can be classified into estrogen response, immune, HRD, and RTK and VEGF. We calculated the ssGSEA score for each pathway using the gsva function from the R package GSVA (version 1.52.3). We retained the log_2_(FPKM+1) score if the biomarker was a molecule. Second, to generate the final estrogen response scores, immune scores, HRD scores, and RTK and VEGF scores presented in Figure 2A, we calculated the mean value of all indicative items across all samples within each subtype.

Second, to generate the final scores presented in Figure 2A, we calculated the mean value of all indicative items across all samples within each subtype as the final score of estrogen response scores, immune scores, HRD scores, and RTK and VEGF scores.

### TME analysis

Gene signatures about immune cell function adapted from Xiao et al. (42) and the cancer-immunity cycle adapted from Mellman et al. (43) were employed for ssGSEA using the gsva function from the R package GSVA (version 1.52.3). This analysis was performed to estimate the abundance of different immune cell types and the function of the antitumor immunity in each sample based on gene expression data.

### IHC

Paraffin-embedded slides were deparaffinized at 60°C for 20 minutes, washed in xylene, and rehydrated in fresh absolute ethanol, 90% ethanol, and 80% ethanol. For H&E staining, the slides were stained with Mayer’s hematoxylin (Sigma-Aldrich) and sodium bicarbonate and counterstained with eosin Y solution (Sigma-Aldrich). IHC staining was performed with primary antibodies against TUBB3 (Abcam, ab215037, 1:500). Slides were visualized under an Olympus BX43 microscope. The IHC results were interpreted by 2 independent pathologists who were previously blinded to the clinicopathological data.

### Immunofluorescence

Immunofluorescence was performed on mouse xenograft sections to detect GABA (Sigma-Aldrich, A2052, 1:1,000). Paraffin-embedded slides were deparaffinized at 60°C for 20 minutes; washed in xylene; rehydrated in fresh absolute ethanol, 90% ethanol, and 80% ethanol; permeabilized in PBS containing 0.2% Triton X-100; and blocked in 1% BSA containing 22.52 mg/mL glycine in PBS-Tween to avoid nonspecific binding of the antibodies. The slides were incubated with the anti-GABA primary antibody (Sigma-Aldrich, A2052, 1:1,000) overnight at 4°C, washed 3 times in PBS, and then incubated with the Alexa Fluor 488–conjugated secondary antibody (Abcam, ab150077, 1:500) for 1 hour at room temperature. Microscopic analyses were performed using a Leica SP5 confocal laser scanning microscope (Leica Microsystems). The stained slides were scanned using a Vectra Polaris System (PerkinElmer) and analyzed using QuPath software (v0.3.2).

### Xenograft mouse models and treatment

RTX (CFW Laboratories Inc., A-125100-NU4) was used to deplete sensory neurons. Female 4-week-old BALB/c nude mice were lightly anesthetized by inhalation of 3% isoflurane (Patterson Veterinary) in oxygen using a precision vaporizer. Three RTX escalating doses (30 μg/kg, 70 μg/kg, 100 μg/kg, diluted in PBS with 1.2% DMSO and 0.06% Tween-80) were subcutaneously injected into the flanks of anesthetized mice on 3 consecutive days, as adapted from established protocols (44). Control littermates were injected with vehicle solution on the same days (PBS with 1.2% DMSO and 0.06% Tween-80). Mice were used for the experiments 4 weeks after the last injection of RTX, and vehicle- and RTX-treated mice were housed together before and during the experiments. The efficiency of RTX treatment in depleting sensory neurons was confirmed by counting the number of CGRP-positive neurons in breast cancer tissue via microscopy. A total of 5 × 10^6^ MCF-7 cells were inoculated into the mammary fat pads of the vehicle- and RTX-treated mice. Tumor sizes were examined every 3 days and calculated as tumor volumes = (length × width^2^)/2 after the tumors were palpable. Once the mice were euthanized, the tumors were removed and weighed.

### Analysis of spatial transcriptomics

#### Visium spot deconvolution.

Robust Cell Type Decomposition with spacexr 2.0.1 (45) in R was used to deconvolve Visium spots into cell types using the unsupervised scRNA-seq reference. The representative cell type for each spot is determined by the cell type with the highest proportion.

#### Cell-cell communication scores for gene pairs based on spatial interaction.

Spatial networks were built using Giotto package (46) (https://giottosuite.com) to connect single cells based on their physical distance to each other. Utilizing Delaunay method, neighbors will be decided by Delaunay triangulation and a maximum distance criterion. After that, ligand-receptor (LR) interactions are scored by using the Delaunay network built above. The desired linkages are extracted from the network and used to calculate the LR interaction score, which measures pairwise LR expression between specified cell types. Linkages are reoriented to differentiate between ligand- and receptor-expressing cells. This score is compared with a distribution of scores produced using randomized linkages to determine whether the given configuration of interactions between specified cell types markedly enriches for pairwise LR expression.

#### CosMx SMI tissue processing and sequencing.

For high-plex in situ transcriptomic profiling, paired baseline and posttreatment FFPE blocks were sectioned (4 μm; 4–5 consecutive sections) and handled under cold-chain conditions until processing. The CosMx SMI technology was utilized for amplification-free, hybridization-based single-molecule barcoding on standard pathology glass slides. The SMI approach employs a 2-component probe design, consisting of a target-binding domain and a readout domain, which is subsequently hybridized with fluorescently labeled branched reporters to enable a 64-bit barcoding scheme through combinatorial signaling across 4 fluorescent channels. The workflow involved standard tissue preparation, followed by probe hybridization and cyclic reporter readout within a flow cell integrated with the SMI instrument. In this study, a comprehensive gene panel encompassing 6,175 targets was utilized. Following each round of reporter hybridization and imaging, a UV cleavage step efficiently quenched the fluorescent signals, allowing for subsequent incubation with the next set of reporters.

#### CosMx SMI data processing and quality control.

For CosMx SMI data, doublets were assessed using the Scrublet function in Scanpy, and cells identified as doublets, along with those within the top 2% of total transcript counts across all cells, were filtered out. Cells with fewer than 60 genes or 100 total transcripts were removed. Cells with a proportion of negative probe counts exceeding 10% were excluded. Additionally, cells with a transcript-to-gene ratio not exceeding 1 were filtered out. Subsequently, the expression values of the filtered cells were normalized based on the total transcript count per cell, followed by log1p transformation.

### Statistics

Efficacy was assessed in the intention-to-treat population, which included all the patients who had undergone randomization. Statistical analyses of clinical outcomes were performed in accordance with the study protocol and the statistical analysis plan (19). Data are presented as mean ± SEM and median and quartiles. A comparison of continuous variables was conducted using the 2-sided Student’s *t* test, Welch’s *t* test, or Wilcoxon’s rank-sum test, depending on the data distribution and variance homogeneity. A comparison of categorical variables was conducted using either the χ^2^ test or Fisher’s exact test. Multiple comparisons were conducted using 1-way and 2-way ANOVA. Spearman’s rank correlation test was used to examine the relationship between 2 variables. A *P* value less than 0.05 was considered significant.

### Study approval

All work with patients was approved by the Ethics Committee in FUSCC (Approval No. 2209261-7). Written informed consent was obtained from all participants prior to their participation. All the authors attest that the trial was conducted in accordance with the protocol and its amendments and with the standards of Good Clinical Practice and the Declaration of Helsinki. The drug manufacturer supplied drugs but played no role in the design of the study, the collection or analysis of the data, or the preparation of the manuscript. A data and safety monitoring board meets periodically. All animal experiments were conducted in accordance with guidelines from the NIH’s *Guide for the Care and Use of Laboratory Animals* (National Academies Press, 2011). Protocols were approved by the Institutional Animal Care and Use Committee of Shanghai Laboratory Animal Center (No. FUSCC-IACUC-2024449).

### Data availability

The WGS, somatic targeted sequencing, and RNA-seq data generated by this study have been submitted to the National Omics Data Encyclopedia (NODE) under accession OEZ00023077 (https://www.biosino.org/node/analysis/detail/OEZ00023077). The data will be made available only upon reasonable request. Requests can be made by completing the application form via the NODE. TCGA, METABRIC, and CPTAC data were downloaded from the cBioPortal website (https://www.cbioportal.org/). The I-SPY2 data were downloaded from NCBI’s Gene Expression Omnibus SuperSeries GSE196096 (https://www.ncbi.nlm.nih.gov/geo/query/acc.cgi?acc=GSE196096). Values for all experimental data points in graphs are reported in the Supporting Data Values file.

## Author contributions

ZMS, YZJ, JJL, and LH participated in the conception and design of the study. YX, FY, YJQ, LC, and HZ generated the data. YX, FY, YJQ, SWZ, HZ, and RS performed the experiments and bioinformatic and statistical analysis. YX, FY, YJQ, HZ, SWZ, and RS wrote, reviewed, and/or revised the manuscript. ZMS, YZJ, JJL, and LH supervised the study. All authors approved the final manuscript.

## Conflict of interest

The authors have declared that no conflict of interest exists.

## Funding support

- National Natural Science Foundation of China, grant numbers U25C2032 and 82441028 to ZMS and 82573645 to YX.
- The Shanghai Key Laboratory of Breast Cancer, grant number 12DZ2260100, to ZMS.
- The Human Phenome Data Center of Fudan University.

## Supplementary Material

Supplemental data

ICMJE disclosure forms

Supplemental tables 1-7

Supporting data values

## Figures and Tables

**Figure 1:**
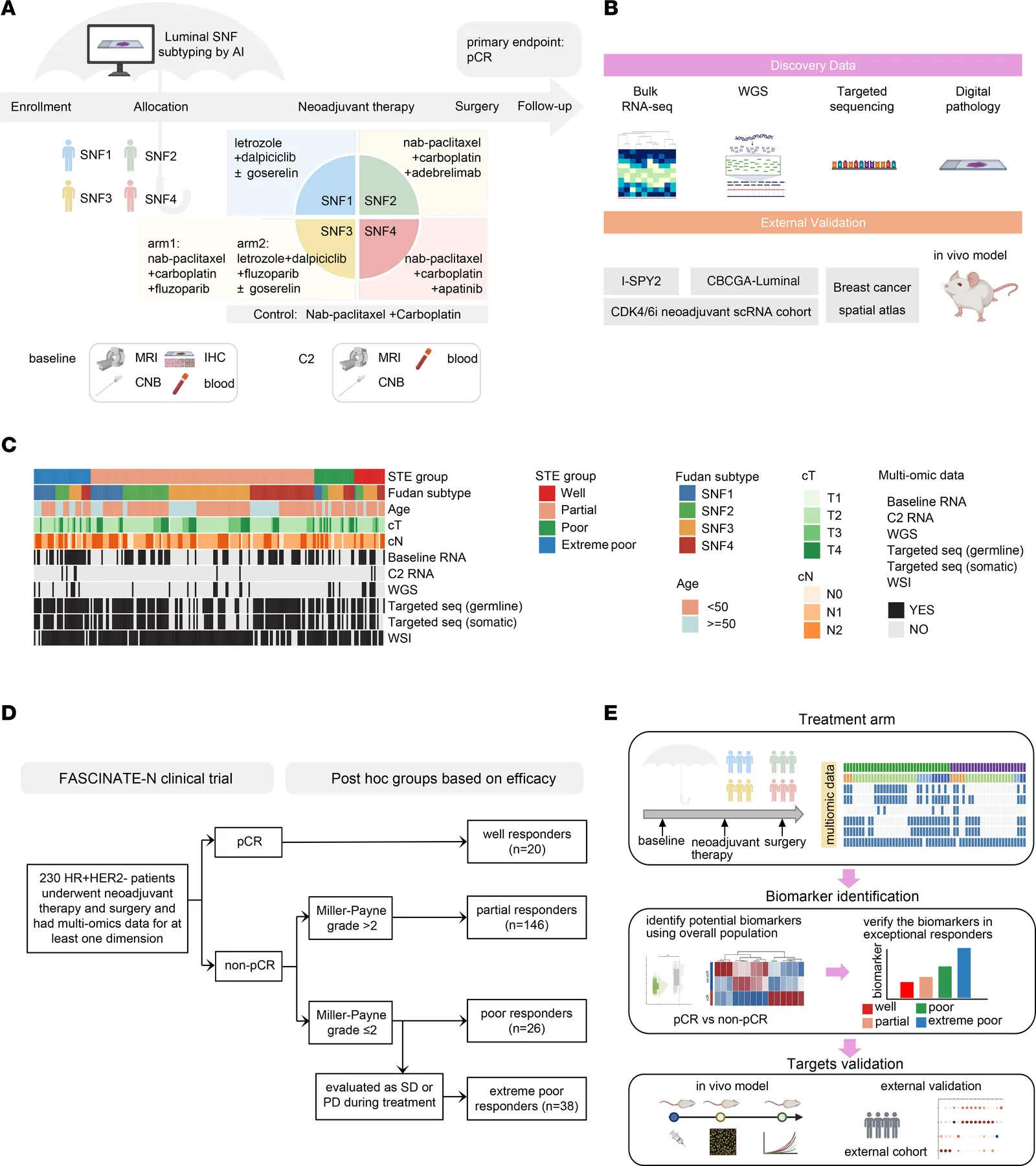
Trial design and data. (**A**) Overall schematic of the HR^+^/HER2^–^ cohort in the FASCINATE-N clinical trial. (**B**) Multiomics data modality of the discovery cohort and external validation strategies. (**C**) Overview of data availability for WGS, RNA-seq, targeted sequencing, and digital pathology across the FASCINATE-N HR^+^/HER2^–^ cohort. (**D**) The definition of exceptional responders. (**E**) Translational design of FASCINATE-N trial. AI, artificial intelligence; cN, clinical node; CNB, core needle biopsy; cT, clinical tumor; CBCGA, Chinese Breast Cancer Genome Atlas; pCR, pathological complete response; PD, progressive disease; scRNA, single-cell RNA; SD, stable disease; STE, special therapeutic effect; WSI, whole-slide imaging; WGS, whole-genome sequencing.

**Figure 2:**
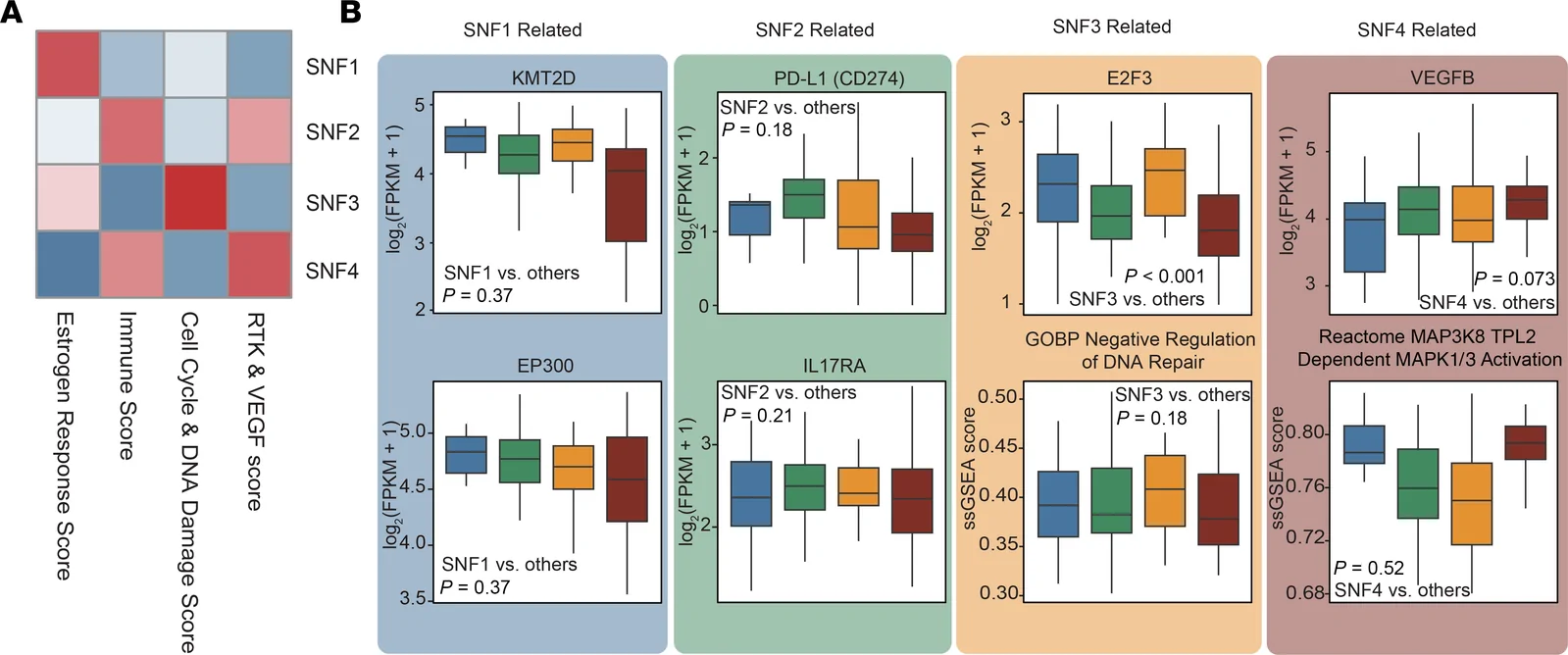
Expression of molecular biomarkers across the FASCINATE-N multiomics cohort. (**A**) Subtype-specific feature scores of specific molecular features in 4 SNF subtypes. ER, estrogen receptor. (**B**) Expression level or single-sample gene set enrichment analysis (ssGSEA) scores of representative biomarkers in 4 SNF subtypes. *P* values were calculated via the Welch’s *t* test. Box plots show the interquartile range, median (line), and minimum and maximum (whiskers). FPKM, fragments per kilobase of transcript per million mapped reads.

**Figure 3:**
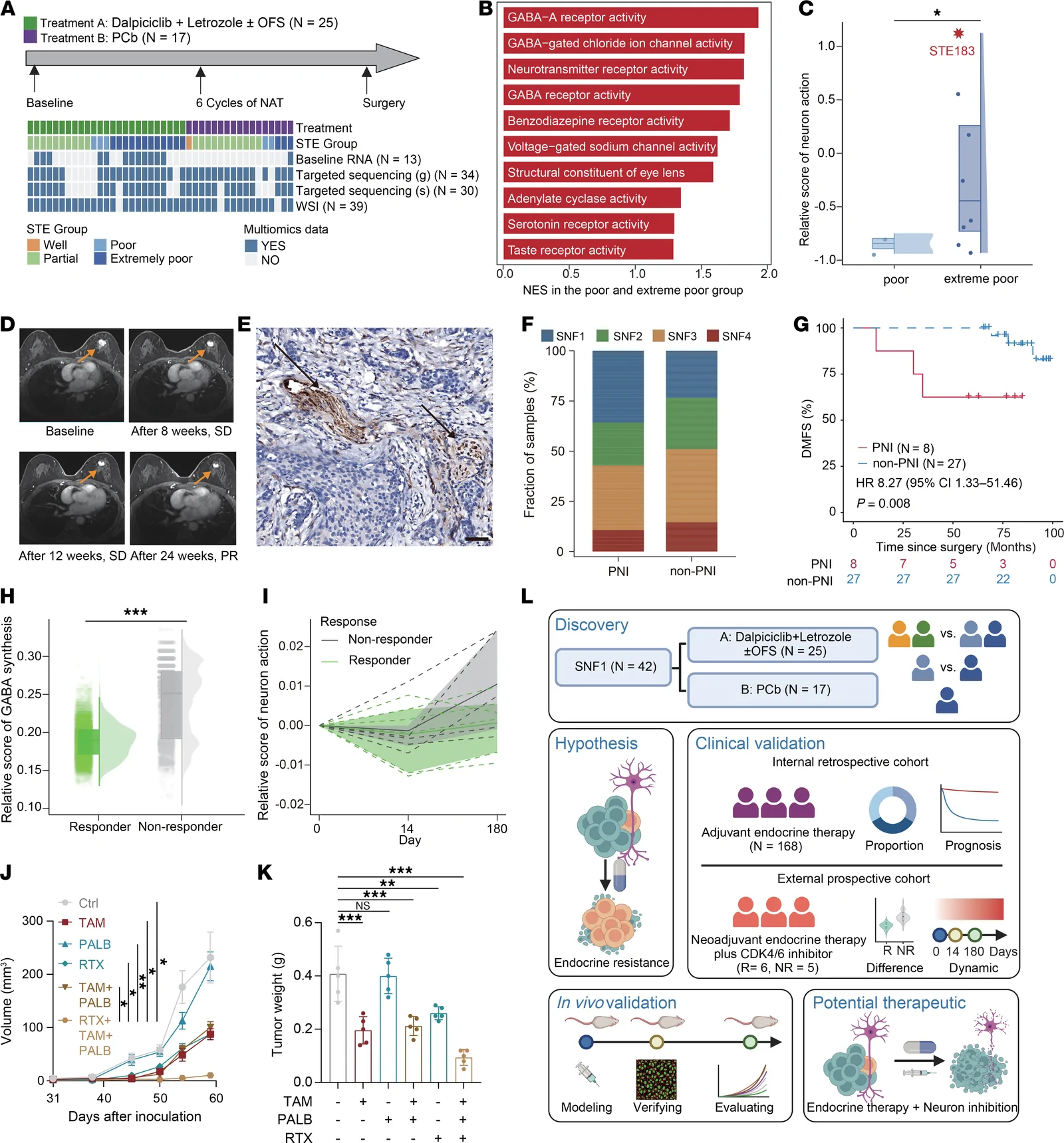
GABAergic neurons undermine the effect of endocrine therapy for SNF1 HR^+^/HER2^–^ breast cancers. (**A**) Overview of enrollment, sequencing, and treatment efficacy of patients with the SNF1 subtype of HR^+^/HER2^–^ breast cancer. (**B**) Upregulated Gene Ontology Molecular Function pathways in the poor and extremely poor patients compared with partial response patients by GSEA. (**C**) Comparison of the relative pathway score of neuron action between patients with poor and extreme poor therapy efficacy in the FASCINATE-N cohort. *P* values were calculated via unpaired Student’s *t* test; *, *P* < 0.05. (**D**) MRI of STE183 at baseline, after 8 weeks, after 12 weeks, and after 24 weeks of neoadjuvant therapy. (**E**) The classical PNI (black arrows) from patient STE183 presented by tubulin-βIII staining. Scale bar, 50 μm. (**F**) Comparison of the proportion of SNF subtypes between patients with or without PNI in the HR^+^/HER2^–^ breast cancer patients from the FUSCC. (**G**) Kaplan-Meier curves of DMFS in SNF1 patients grouped according to the existence of PNI from the FUSCC (*N* = 35). (**H** and **I**) Relative pathway scores of GABA synthesis (**H**) and neuron action (**I**) in responders vs. nonresponders to endocrine therapy plus CDK4/6 inhibitor (GSE158724). *P* values were calculated via unpaired Student’s *t* test; ***P < 0.001. (**J** and **K**) Tumor growth curves (**J**) and final tumor weights (**K**) of the MCF7 orthotopic tumors in BALB/c nude mice treated with RTX, TAM, and PALB (*N* = 5). *P* values were calculated via 2-way (**J**) and 1-way (**K**) ANOVA; *, *P* < 0.05; **, *P* < 0.01; ***, *P* < 0.001. (**L**) Schematic overview of the analysis of SNF1 arm. Box plots show the interquartile range, median (line), and minimum and maximum (whiskers). OFS, ovarian function suppression; NAT, neoadjuvant therapy; NES, normalized enrichment score; WSI, whole-slide imaging; SD, stable disease; PR, partial response; PNI, perineural invasion; DMFS, distant metastasis–free survival; TAM, tamoxifen; PALB, palbociclib; RTX, resiniferatoxin.

**Figure 4:**
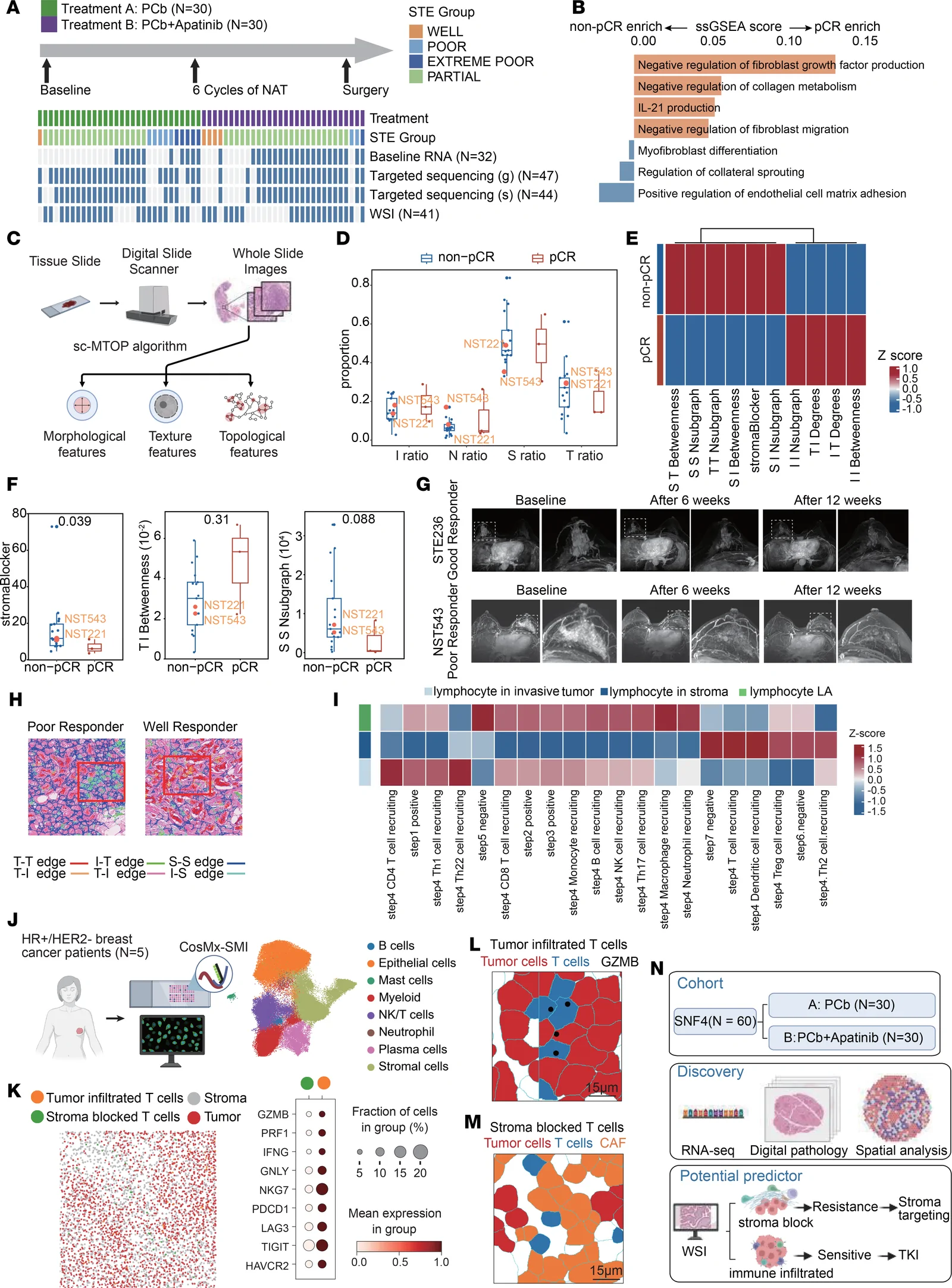
Spatial structures influence the efficacy of apatinib in SNF4 HR^+^/HER2^–^ breast cancers. (**A**) Overview of enrollment, sequencing, and treatment efficacy of patients with the SNF4 subtype of HR^+^/HER2^–^ breast cancer. (**B**) Box plot showing the relative pathway ssGSEA scores for pathways between pCR and non-pCR patients. (**C**) Workflow for extracting morphological, texture, and topological features of WSIs from baseline biopsy using sc-MTOP. (**D**) Comparisons of cell type proportions extracted from WSIs between pCR and non-pCR patients, with exceptional nonresponders highlighted. *P* values were calculated via unpaired Student’s *t* test. (**E**) Heatmap showing scaled pathological feature expression between the pCR and non-pCR patients. (**F**) Bar plot comparing the topological features between pCR and non-pCR patients, with exceptional nonresponders highlighted. *P* values were calculated via unpaired Student’s *t* test. (**G**) MRI of STE236 and NST543 at baseline, after 6 weeks, and after 12 weeks of neoadjuvant therapy. (**H**) Representative WSIs showing the topological features between 2 exceptional responders (STE236 and NST543), with different colored lines representing different types of cell connections. (**I**) Heatmap showing immune cycle pathways in lymphocytes across tissue regions. (**J**) Design and cellular landscape of CosMx-SMI profiling. Tumor samples from 5 HR^+^/HER2^–^ breast cancer patients were analyzed by CosMx-SMI, identifying major cell populations. (**K**) Cytotoxic effector gene expression in tumor-infiltrated versus stroma-restricted T cells. (**L** and **M**) Representative images showing the GZMB expression in T cells adjacent to tumor cells or cancer-associated fibroblasts. (**N**) Schematic overview of the analysis of SNF4 arm. Box plots show the interquartile range, median (line), and minimum and maximum (whiskers). PCb, nab-paclitaxel, carboplatin; NAT, neoadjuvant therapy; I, immune cells; N, normal cells; S, stroma cells; T, tumor cells; SD, stable disease; PD, progressive disease; WSI, whole-slide image; GZMB, granzyme B; CAF, cancer-associated fibroblast.

**Figure 5:**
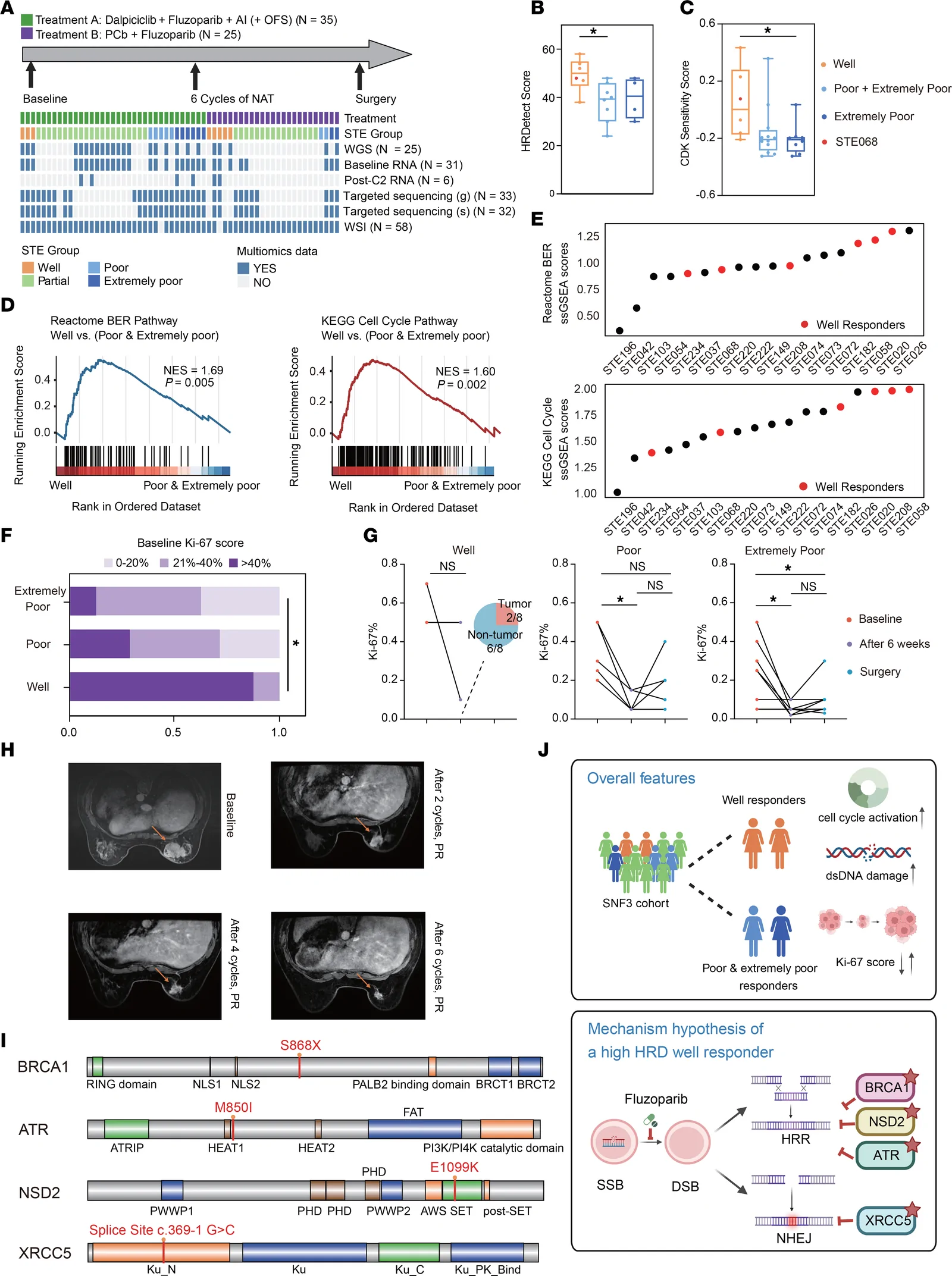
Determinants of exceptional responses to PARP inhibitors combined with CDK4/6 inhibitors in SNF3 HR^+^/HER2^–^ breast cancers. (**A**) Overview of enrollment, sequencing, and treatment efficacy of patients with the SNF3 subtype of HR^+^/HER2^–^ breast cancer. (**B** and **C**) Baseline HRD scores based on HRDetect algorithm (**B**) and CDK4/6 inhibitor sensitivity scores based on ssGSEA (**C**) of exceptional responders. *P* values were calculated via 1-way ANOVA; *, *P* < 0.05. Box plots show the interquartile range, median (line), and minimum and maximum (whiskers). (**D**) GSEA results of KEGG cell cycle pathway and Reactome BER pathway, well vs. poor and extreme poor responders. (**E**) Ranked ssGSEA scores for the Reactome BER and KEGG cell cycle pathways. Samples were ordered in ascending order of their scores, with well responders highlighted in red. (**F**) Fraction of baseline Ki-67 scores of exceptional responders based on core needle biopsy. *P* values were calculated via χ^2^ test; *, *P* < 0.05. (**G**) Ki-67 scores at baseline, after 6 weeks, and at surgery among the well, poor, and extreme poor responders. The pie chart illustrates that complete tumor eradication was observed in 6 out of 8 well responders after 2 cycles of neoadjuvant therapy. *P* values were calculated via paired Student’s *t* test; *, *P* < 0.05. (**H**) MRI of STE068 at baseline, after 2 cycles, after 4 cycles, and after 6 cycles of neoadjuvant therapy. (**I**) Mutation information of genes related to double-stranded DNA repair deficiency of STE068, including BRCA1, ATR, NSD2, and XRCC5. (**J**) Schematic overview of the analysis of SNF3 arm. NAT, neoadjuvant therapy; DSB, double-strand break; SSB, single-strand break; PARP, poly (ADP-ribose) polymerase; OFS, ovarian function suppression; HRD, homologous recombination deficiency; BER, base excision repair; KEGG, Kyoto Encyclopedia of Genes and Genomes.

**Figure 6:**
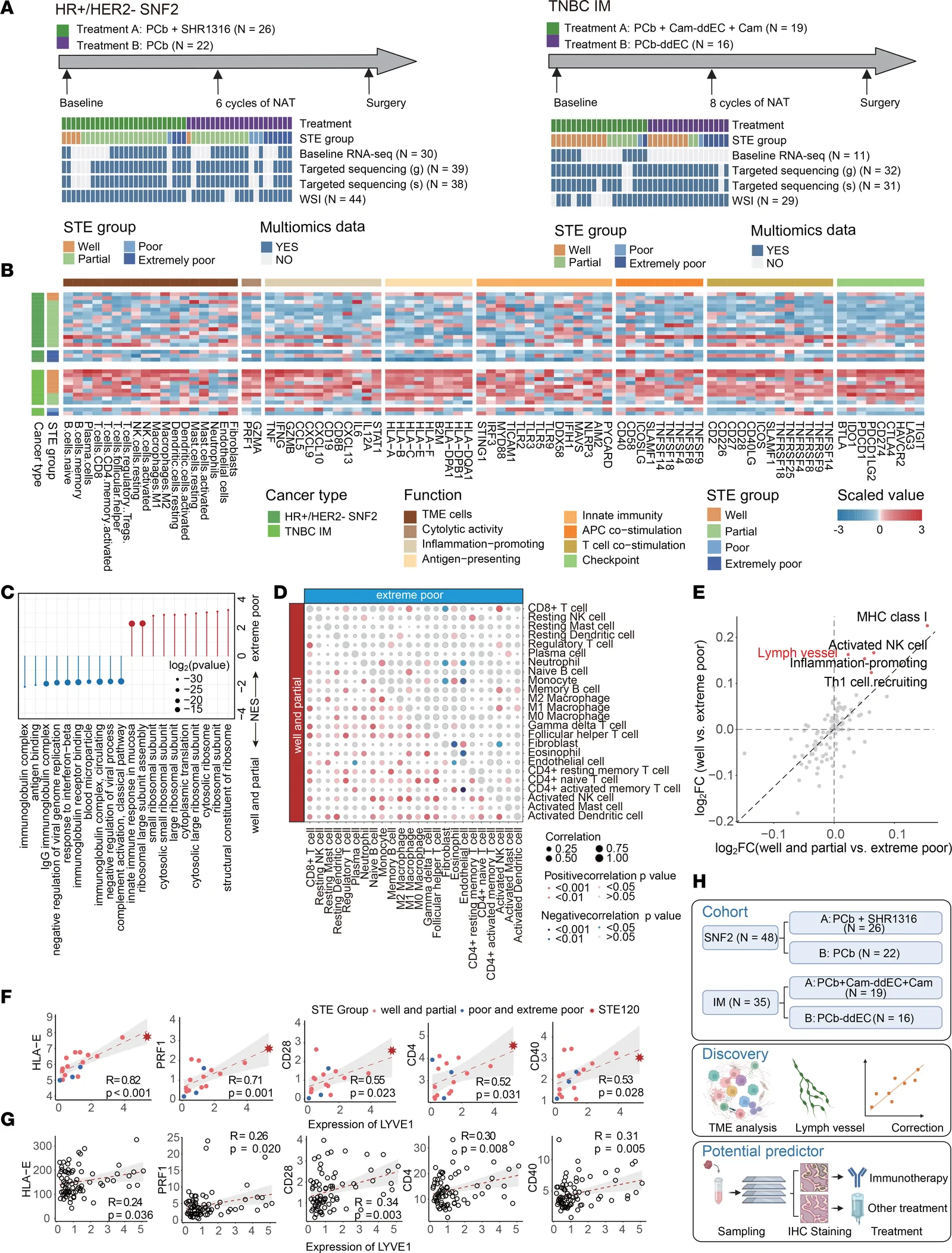
Key immune response patterns and predictive markers in SNF2 HR^+^/HER2^–^ breast cancers. (**A**) Overview of enrollment, treatment, sequencing, and treatment efficacy of SNF2 subtype from HR^+^/HER2^–^ breast cancer and IM subtype from TNBC. (**B**) Heatmaps showing the immune landscapes of tumor samples at baseline from patients in the HR^+^/HER2^–^ SNF2 experimental arm and in the TNBC IM experimental arm. The treatment arm and response were annotated on the left. (**C**) The NESs of the pathways at the transcriptomic level of the favorable- (well and partial) group versus (extreme poor) unfavorable-efficacy group. (**D**) Correlations among cell types in the favorable-efficacy group (lower left panel) and unfavorable-efficacy group (upper right panel). (**E**) Log_2_ fold-changes of the immune signature scores between favorable- and unfavorable-efficacy groups from the whole cohort (*x* axis) and the exceptional responders (*y* axis). The sources of the immune signatures are summarized in the Methods section. (**F**) Spearman’s correlations between the mRNA levels of *LYVE1* and the genes encoding immune-related molecules in SNF2 patients from FASCINATE-N cohort. Shaded areas represent 95% confidence intervals. (**G**) Spearman’s correlations between the mRNA levels of *LYVE1* and the genes encoding immune-related molecules in SNF2 patients from CBCGA cohort. Shaded areas represent 95% confidence intervals. (**H**) Schematic overview of the analysis of SNF2 arm. NAT, neoadjuvant therapy; IM, immunomodulatory; TNBC, triple-negative breast cancer; CBCGA, Chinese Breast Cancer Genome Atlas.

## Version 1 10/15/2026

Electronic publication
