# Supplementary material for: Multimodal assessment of exceptional responders refines subtyping-directed precision treatment in HR+/HER2– breast cancer
Source: J Clin Invest. 2026 Oct 15;136(20):e202305. doi: 10.1172/JCI202305 (PMC13668612; doi:10.1172/JCI202305)
Supplement: Supplementary file 1 [file jci-136-202305-s098.pdf]

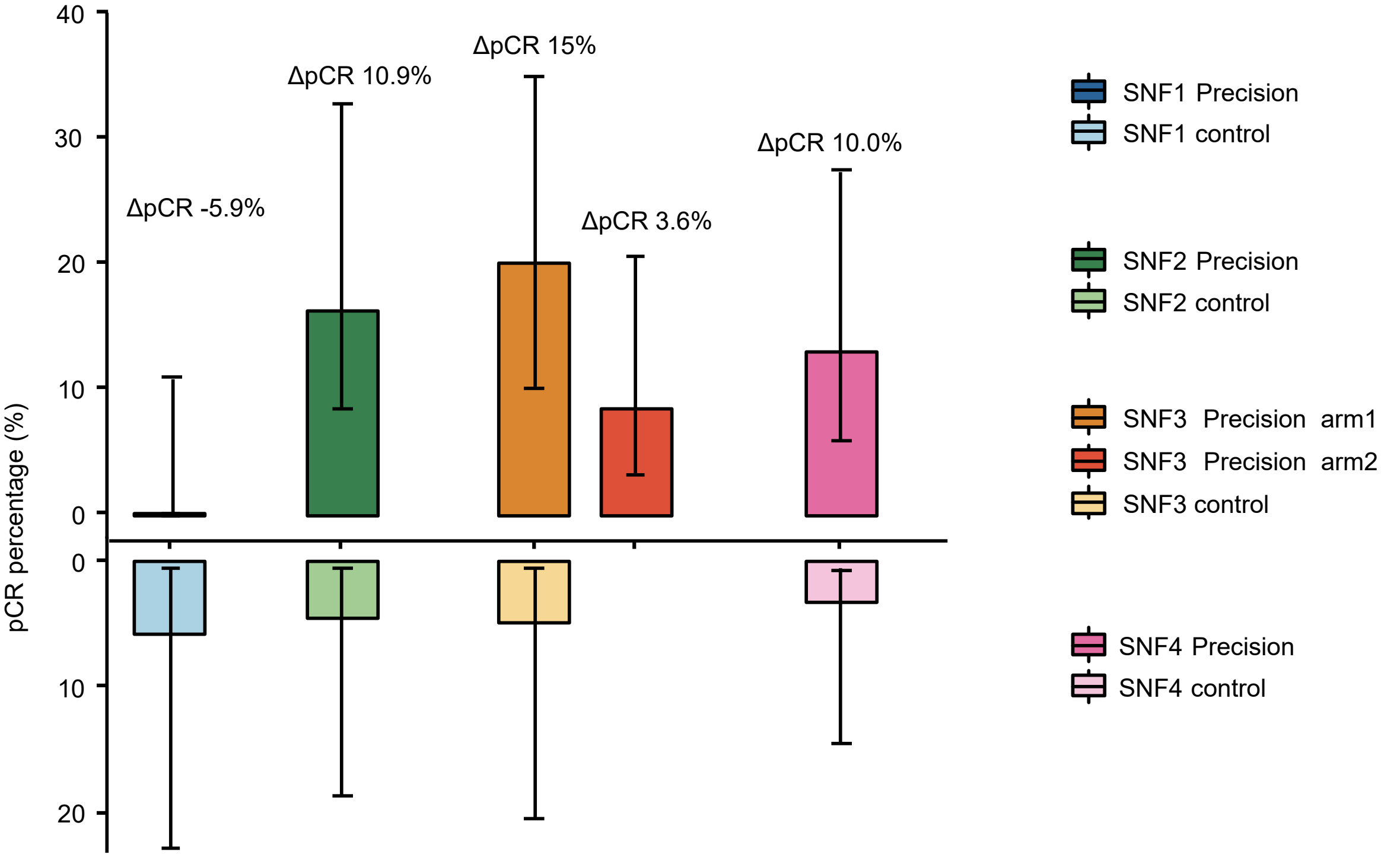

Supplementary Figure 1. Pathological complete response rates in 230 patients with HR+/ HER2- breast cancer.

Boxplot showing the pathological complete response rates and 90% confidence interval in each SNF precision arm and control arm in the intention-to-treat population.

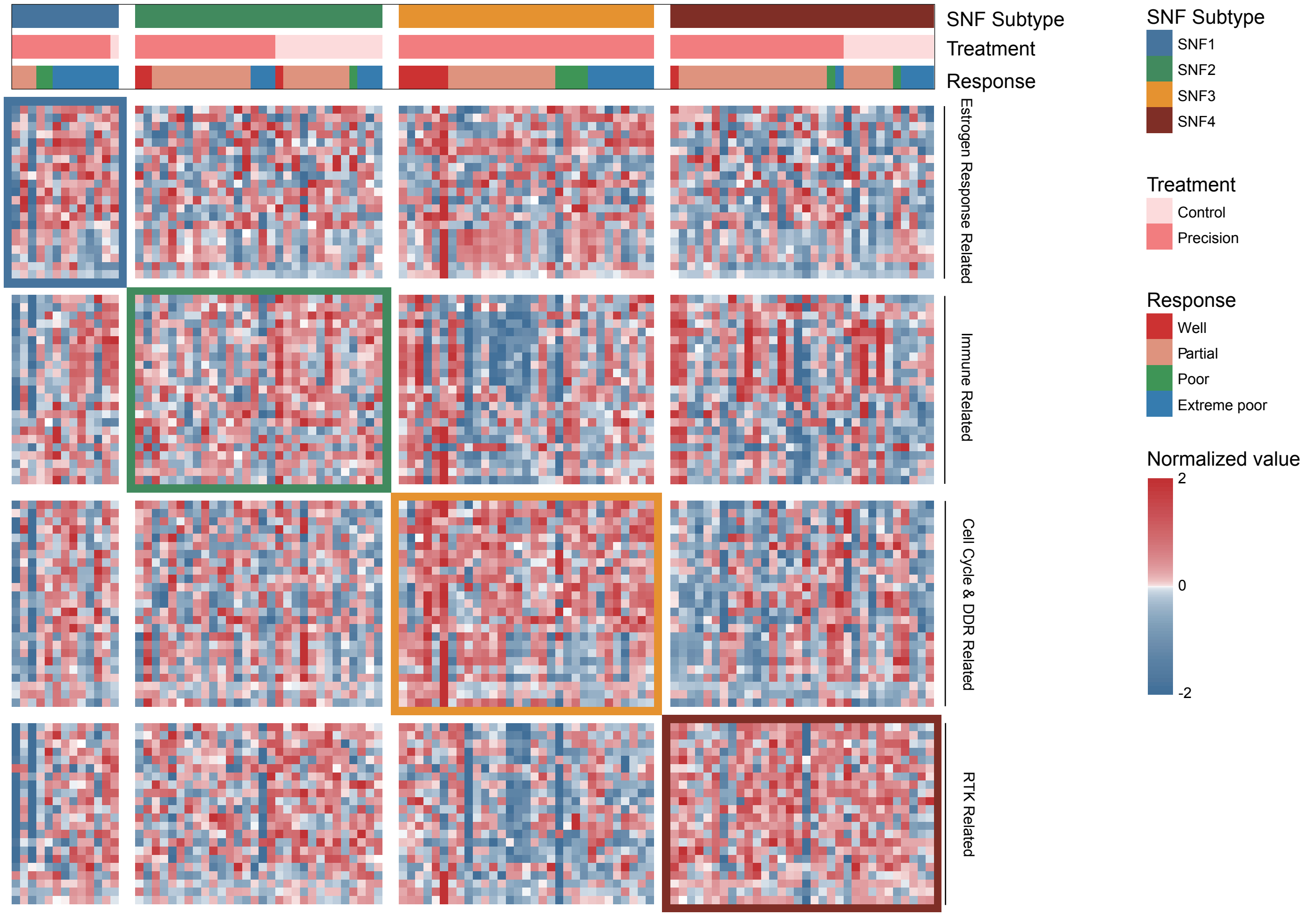

Supplementary Figure 2. RNA-based biomarkers validate AI-based precision subtyping.

Heatmap showing the normalized ssGSEA scores of pathways or normalized $\log_2$(FPKM+ 1) values of genes reflecting intrinsic characteristics of four AI-based SNF subtypes.

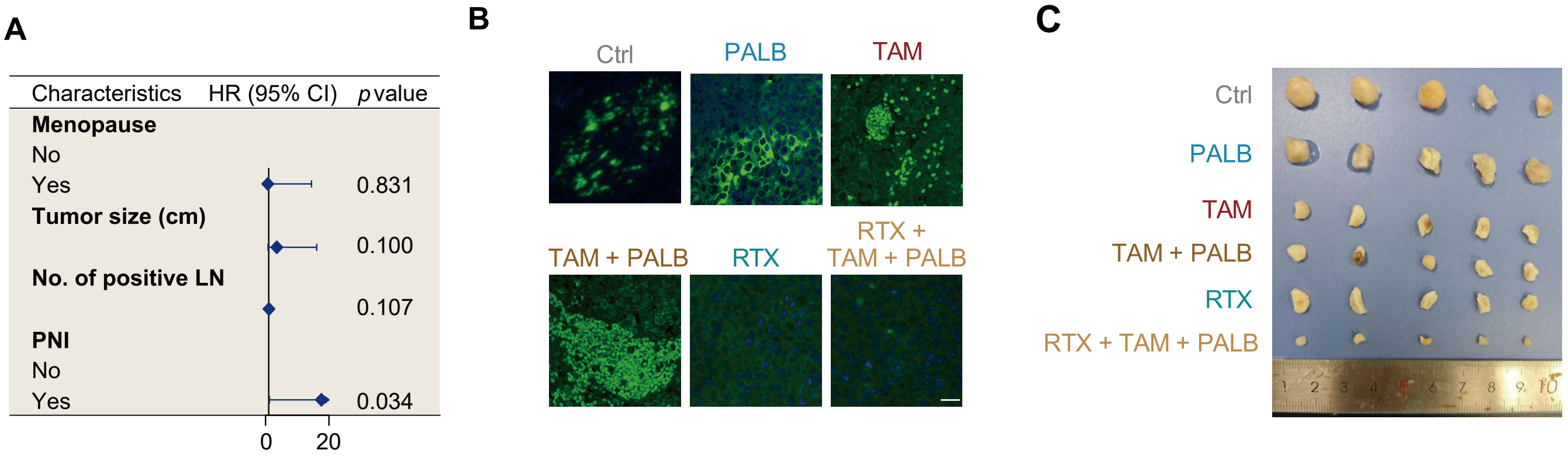

Supplementary Figure 3. GABAergic neurons impair the response to endocrine therapy in SNF1 HR+/HER2− breast cancer.

(A) Multivariate analysis of the PNI for DMFS in the FUSCC-SNF1 cohort (N = 35). (B-C) GABA staining (B) and tumors (C) from the orthotopic MCF7 tumor model in BALB/c nude mice treated with RTX, TAM, and PALB (corresponding to Figure 3K). Scale bar, 20 μm. Data are representative of 3 independent experiments in Supplementary Figure 3B.

Abbreviations: CI, confidence interval; PNI, perineural invasion; TAM, tamoxifen; PALB, Palbociclib; RTX, resiniferatoxin.

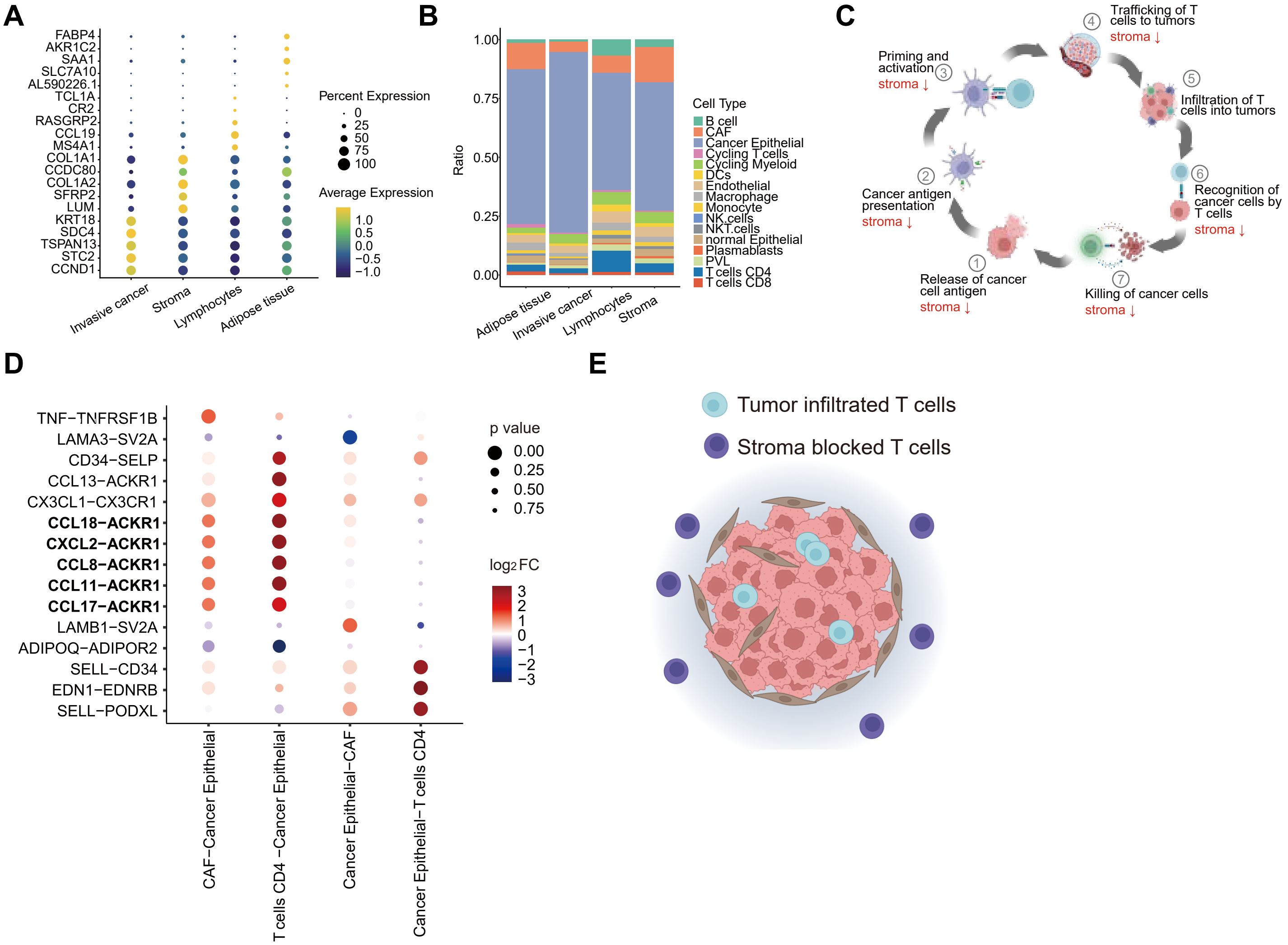

Supplementary Figure 4. Spatial characteristics and potential mechanism of exceptional responders.

(A) Dot plot showing the top differentially expressed genes across different tissue regions. (B) Bar plot showing the proportions of cell composition across different tissue regions. (C) Schematic illustration of immune cycle pathways and changes in stromal regions. (D) Dot plot showing the interactions between different cell types. (E) Representative spatial map of T-cell distribution.

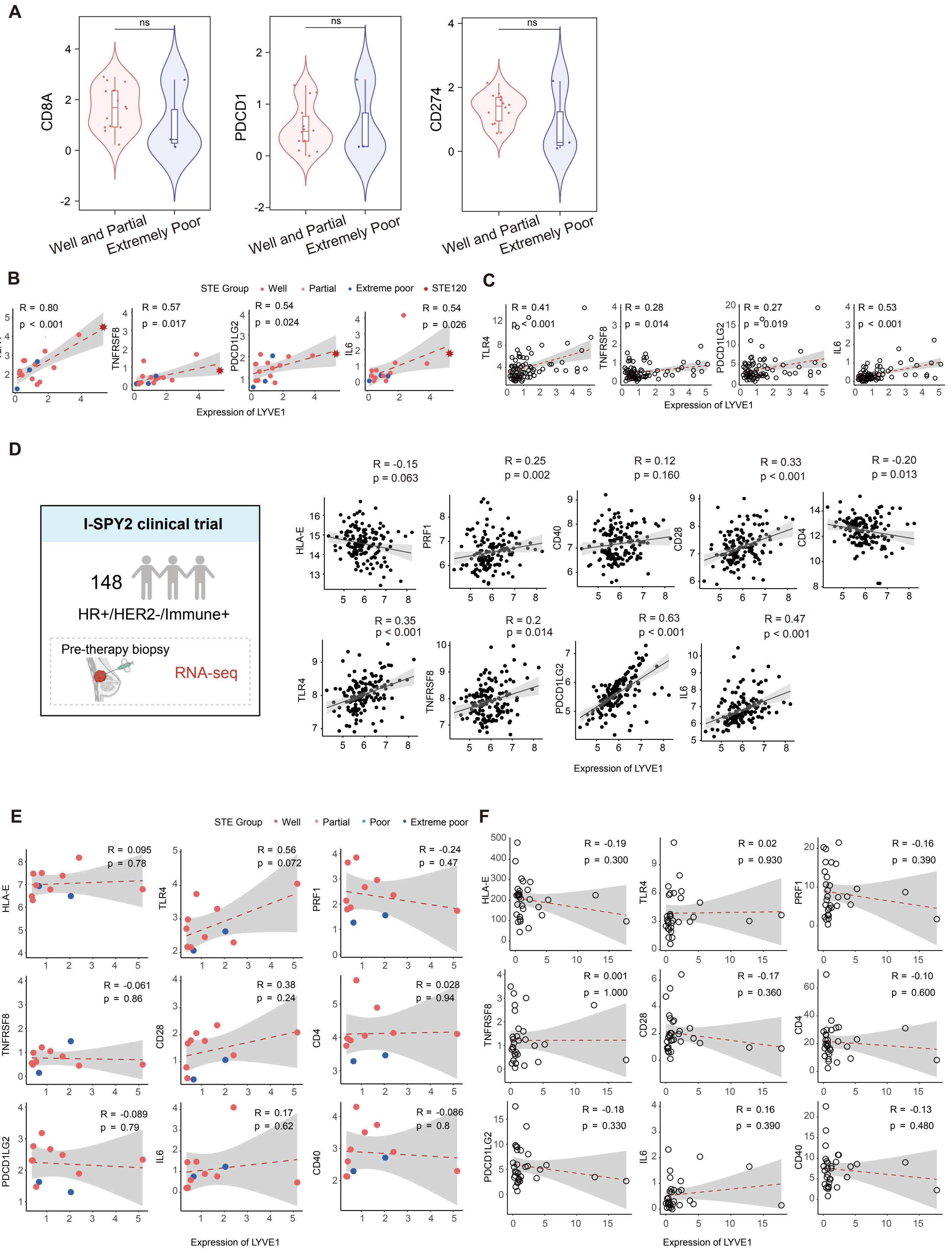

A
ns
CD8A
PDCD1
CD274
Well and Partial
Extremely Poor
B
STE Group
Well
Partial
Extreme poor
STE120
R = 0.80
p < 0.001
TLR4
R = 0.57
p = 0.017
TNFRSF8
R = 0.54
p = 0.024
PDCD1LG2
R = 0.54
p = 0.026
IL6
Expression of LYVE1
C
R = 0.41
p < 0.001
TLR4
R = 0.28
p = 0.014
TNFRSF8
R = 0.27
p = 0.019
PDCD1LG2
R = 0.53
p < 0.001
IL6
Expression of LYVE1
D
I-SPY2 clinical trial
148
HR+/HER2-/Immune+
Pre-therapy biopsy
RNA-seq
R = -0.15
p = 0.063
HLA-E
R = 0.25
p = 0.002
PRF1
R = 0.12
p = 0.160
CD40
R = 0.33
p < 0.001
CD28
R = -0.20
p = 0.013
CD4
R = 0.35
p < 0.001
TLR4
R = 0.2
p = 0.014
TNFRSF8
R = 0.63
p < 0.001
PDCD1LG2
R = 0.47
p < 0.001
IL6
Expression of LYVE1
E
STE Group
Well
Partial
Poor
Extreme poor
R = 0.095
p = 0.78
HLA-E
R = 0.56
p = 0.072
TLR4
R = -0.24
p = 0.47
PRF1
R = -0.061
p = 0.86
TNFRSF8
R = 0.38
p = 0.24
CD28
R = 0.028
p = 0.94
CD4
R = -0.089
p = 0.79
PDCD1LG2
R = 0.17
p = 0.62
IL6
R = -0.086
p = 0.8
CD40
Expression of LYVE1
F
R = -0.19
p = 0.300
HLA-E
R = 0.02
p = 0.930
TLR4
R = -0.16
p = 0.390
PRF1
R = 0.001
p = 1.000
TNFRSF8
R = -0.17
p = 0.360
CD28
R = -0.10
p = 0.600
CD4
R = -0.18
p = 0.330
PDCD1LG2
R = 0.16
p = 0.390
IL6
R = -0.13
p = 0.480
CD40
Expression of LYVE1

Supplementary Figure 5. Key immune response patterns and predictive markers in SNF2 tumors distinct from TNBC.

(A) Comparison of classical biomarkers for immunotherapy efficacy between Well and partial group and Extreme poor group in the FASCINATE-N cohort. P values were calculated via the Student's t-test. (B-F) Spearman correlations between *LYVE1* mRNA expression and the expression of genes encoding immune-related molecules in patients with SNF2 tumors from the FASCINATE-N cohort (B), patients with SNF2 tumors from the CBCGA cohort (C), patients with HR+/HER2-/immune+ tumors from the I-SPY2 cohort (D), patients with IM tumors from the FASCINATE-N cohort (E), and patients with IM tumors from the CBCGA cohort (F). Shaded areas represent 95% confidence intervals.
